# Probing the multimodal fungiform papilla: complex peripheral nerve endings of chorda tympani taste and mechanosensitive fibers before and after Hedgehog pathway inhibition

**DOI:** 10.1007/s00441-021-03561-1

**Published:** 2021-12-03

**Authors:** Christopher R. Donnelly, Archana Kumari, Libo Li, Iva Vesela, Robert M. Bradley, Charlotte M. Mistretta, Brian A. Pierchala

**Affiliations:** 1 Department of Biologic and Materials Sciences, University of Michigan School of Dentistry, Ann Arbor, MI USA; 2 Center for Translational Pain Medicine, Department of Anesthesiology, Duke University Medical Center, Durham, NC USA; 3 Duke Cancer Institute, Duke University Medical Center, Durham, NC USA; 4 Rowan University School of Osteopathic Medicine, Stratford, NJ USA; 5 Department of Anatomy, Cell Biology & Physiology, Stark Neurosciences Research Institute, Indiana University School of Medicine, IN Indianapolis, USA

**Keywords:** Fungiform papilla, Chemosensory, Oral mechanoreception, Sonidegib, Geniculate ganglion, Chorda tympani, Hedgehog pathway inhibition

## Abstract

The fungiform papilla (FP) is a gustatory and somatosensory structure incorporating chorda tympani (CT) nerve fibers that innervate taste buds (TB) and also contain somatosensory endings for touch and temperature. Hedgehog (HH) pathway inhibition eliminates TB, but CT innervation remains in the FP. Importantly, after HH inhibition, CT neurophysiological responses to taste stimuli are eliminated, but tactile responses remain. To examine CT fibers that respond to tactile stimuli in the absence of TB, we used *Phox2b-Cre;* Rosa26^LSL−TdTomato^ reporter mice to selectively label CT fibers with TdTomato. Normally CT fibers project in a compact bundle directly into TB, but after HH pathway inhibition, CT fibers reorganize and expand just under the FP epithelium where TB were. This widened expanse of CT fibers coexpresses Synapsin-1, β-tubulin, S100, and neurofilaments. Further, GAP43 expression in these fibers suggests they are actively remodeling. Interestingly, CT fibers have complex terminals within the apical FP epithelium and in perigemmal locations in the FP apex. These extragemmal fibers remain after HH pathway inhibition. To identify tactile end organs in FP, we used a K20 antibody to label Merkel cells. In control mice, K20 was expressed in TB cells and at the base of epithelial ridges outside of FP. After HH pathway inhibition, K20 + cells remained in epithelial ridges but were eliminated in the apical FP without TB. These data suggest that the complex, extragemmal nerve endings within and disbursed under the apical FP are the mechanosensitive nerve endings of the CT that remain after HH pathway inhibition.

**Supplementary information:**

The online version contains supplementary material available at 10.1007/s00441-021-03561-1.

## Introduction

The anterior mammalian tongue is an exquisitely sensitive structure that has specialized receptors for taste and somatosensation and is adapted to perform motor functions in speaking and eating (Hiiemae and Palmer 2003; Mistretta and Bradley 2021; Todrank and Bartoshuk 1991). The immediate, contemporaneous sensations of taste, touch, and temperature when food contacts the tongue are necessary for nutrient detection and for the rejection of spoiled or potentially poisonous foods. This peripheral recognition of stimuli is transmitted centrally via sensory afferents and is transformed into flavor perception in brain circuits. Classically, the lingual chemosensory receptor organs, the taste buds, and their innervation have been regarded as generally distinct from somatosensory receptors for tactile and thermal sensations. The working paradigm for the anterior tongue had been that taste receptor cells are in taste buds (TB) in fungiform papillae (FP) and are innervated by chorda tympani (CT) nerve fibers from neurons of the geniculate ganglion. In concert, nerve endings and receptors for touch and temperature were considered to be mainly in filiform (FILIF), non-taste papillae, and in epithelial walls of the FP, innervated by lingual nerve (LN) fibers from neurons of the trigeminal ganglion.

CT fibers, however, respond robustly to tongue stimulation with not just chemicals, but also respond to tactile and cold stimuli (Finger et al. 2005; Kumari et al. 2015; Ogawa et al. 1968; Shimatani et al. 2002). Thus, the CT innervates multimodal receptors and/or includes endings that are chemosensory and somatosensory. Likewise, the geniculate ganglion neurons comprising the CT soma are multimodal in their response profiles and receptive field maps (Breza et al. 2006; Yokota and Bradley 2016, 2017). If rodents are treated with the Hedgehog (HH) pathway inhibitor, sonidegib, used in cancer patients, the TB and neurophysiological responses from the CT to chemical stimulation are eliminated (Kumari et al. 2015, 2017, 2018). It is noteworthy, though, that whereas TB are lost, the FP innervation from the CT remains, and responses to tongue stroking and cold-water stimuli are retained (Kumari et al. 2015, 2017; (Mistretta and Kumari 2019). This rodent model involving HH pathway inhibition, therefore, has revealed that TB are not required to sustain the nerve endings of the CT fibers, or the CT fibers synapsing/associating with potential end organs that respond to tactile and cold stimuli. Collectively these studies have amended the classic archetype of the FP as solely a taste papilla, and in a re-conceptualization present a lingual papilla organ that, through CT innervation of geniculate neurons alone, is multimodal for taste, touch, and temperature sensation (Mistretta and Bradley 2021).

To investigate the fiber distributions of oral sensory geniculate ganglion neurons that respond to somatosensory and temperature stimuli, we utilized *Phox2b*-Cre; Rosa26^LSL−TdTomato^ reporter mice in which CT fibers in FP are labeled with tomato/RFP (Donnelly et al. 2017; Ohman-Gault et al. 2017). *Phox2b* (paired-like homeobox 2b) is a transcription factor critical for viscerosensory and autonomic pathway development (D'Autreaux et al. 2011; Pattyn et al. 1999) and is expressed by all CT fibers from geniculate ganglion neurons (Ohman-Gault et al. 2017). We also took advantage of the HH pathway inhibition paradigm, in treating *Phox2b*-Cre; Rosa26^LSL−TdTomato^ mice, hence forth called *Phox2b*-Cre; TdTomato mice, by oral gavage with the smoothened antagonist sonidegib, to eliminate TB and CT chemical taste responses, with retention of CT somatosensory responses (Kumari et al. 2015). A careful morphologic analysis of RFP + fibers allowed for the investigation of CT fibers and nerve endings in the anterior tongue that respond to somatosensory stimuli. We studied innervation to FP and TB after vehicle or sonidegib treatment, and determined the morphological characteristics of the CT nerves in dual labeling experiments with antibodies to neuron/nerve-associated markers, RFP *(Shh* and *Phox2b)*, P2X3, β-tubulin, synapsin, S100, GAP43, NFH, NFL, and K20. The data reveal an extensive, complex innervation within the FP epithelium, in the apical perigemmal epithelium, and in the stroma of the FP, in addition to the CT fibers that project directly into the TB. After HH pathway inhibition in *Phox2b*-Cre; TdTomato mice, we found that RFP + fibers extended beyond their usual spatial distribution in the apical FP, from a compact fiber bundle projecting into the TB to a broader, disbursed bundle under the epithelium where the TB had been located. Furthermore, a highly complex network of CT fiber endings within the papilla apical, perigemmal epithelium, outside of the TB location, appeared to remain largely unaffected after HH pathway inhibition. However, cells exhibiting immunolabeling for keratin 20 (K20), a putative Merkel cell marker, were largely eliminated following HH pathway inhibition, and the few remaining K20 + cells were part of remnant K8 + TB cells. Collectively, the data presented here demonstrate that mechanosensory nerve terminals from the CT are remarkably complex and likely are not substantially affected by TB loss in the context of HH pathway inhibition.

## Materials and methods

### Animals

All animal use and care procedures were performed in accordance with the guidelines of the National Institutes of Health and approved protocols of the University of Michigan and Indiana University Institutional Animal Care and Use Committees (IACUCs). Reporter mice were produced by breeding *Phox2b*-Cre^tg/+^ mice (Jackson Laboratories, strain #016223) with Rosa26^LSL−TdTomato/+^ mice (henceforth, referred to as *Phox2b*-Cre; TdTomato mice; Jackson Laboratories, strain #007909), as we previously reported (Donnelly et al. 2017). For experiments not involving *Phox2b*-Cre*;* TdTomato reporter mice, wild-type littermate mice or C57BL/6 J mice were utilized (Jackson Laboratories, strain #000664). Adult female mice (8 weeks to 6 months old) were used in all experiments. For pharmacologic HH pathway inhibition in *Phox2b*-Cre*;*TdTomato mice, the animals were treated for 25-28d with daily oral gavage of sonidegib [NVP-LDE225 diphosphate salt, ChemieTek, Catalog # CT-LDE225] dissolved in vehicle, PEG 400/5% dextrose in water (75:25 v/v), at a dose of 20 mg/kg, or with vehicle alone. Eleven *Phox2b*-Cre*;*TdTomato mice were gavaged with sonidegib; ten mice were gavaged with vehicle. In addition, in mice that were not *Phox2b*-Cre-positive, to study K8 localizations compared with various other antibodies, two mice were gavaged with vehicle and two with sonidegib for 36 days.

In genetic models that block smoothened (Smo), we used cryopreserved tongues from mice generated to conditionally delete (doxycycline-regulated) Smo globally (R26M2rtTA/ + ;tetO-cre;*Smo*^fl/fl^) or in the epithelium (K5rtTA;tetO-cre;*Smo*^fl/fl^) for 24 days, investigated and reported in a previous study and publication (Kumari et al. 2017). Tongues from three control (littermates negative for rtTA and/or tetO-cre) and three *Smo* deletion mice were analyzed. For investigating *Shh* in nerves, we used cryopreserved tongues from the *ShhCreER;TdTomato* model that received 400 mg tamoxifen/kg in Teklad Global Diet (Harlan) for 30 days as reported in (Kumari et al. 2017).

### Tissue preparation

At the end of HH pathway inhibition treatment, or after recordings from the CT nerve (described in 6), *Phox2b*-Cre*;*TdTomato mice were euthanized and tissues were collected and prepared for analysis, with histology and immunostaining performed as described previously (Kumari et al. 2015). Tongues on mandibles were fixed for 2 h at 4°C in 4% paraformaldehyde in PBS. Tongues were subsequently dissected from the mandible, cut at the more anterior level of the intermolar eminence, and returned to fixative for an additional 2–3 h at 4°C. Tongues were then washed with PBS, trimmed to exclude circumvallate and foliate papillae, and bisected at the midline into two halves. Tissue pieces were cryoprotected overnight with 30% sucrose in PBS followed by embedding in O.C.T. compound (Tissue-Tek, Sakura Finetek). Serial sagittal sections were cut at 6–10 μm, 20 or 30 μm, mounted onto glass slides, and processed for immunostaining.

### Immunostaining and microscopy

Tongue sections were air dried, rehydrated, and blocked for 1–4 h at room temperature in 10% normal donkey serum in PBS-X (0.3% Triton-X100 in PBS, pH 7.4), followed by incubation overnight at 4 °C with primary antibodies. On the following day, slides were washed three times in PBS, followed by incubation with the appropriate secondary antibodies for 1–2 h at room temperature. Sections were mounted after rinsing with PBS in a medium containing DAPI. Primary and secondary antibodies used in this study are listed in Table 1. Imaging was performed using a Nikon Eclipse 80i microscope with a Nikon DS Ri2 camera system and NIS software. Confocal microscopy was performed with a Nikon A1 B high sensitivity microscope. Figures were assembled with Adobe Photoshop, and any adjustments made for brightness and contrast were performed in parallel across all images for any one figure.

**Table 1:** Description of primary and secondary antibodies used in this study

Antibody	Dilutions	Source	Cat. #	Lot #
**Primary antibodies**				
Rabbit anti-RFP	1:1000	Rockland	600–401-379	37,250
Goat anti-RFP	1:2000	MyBioSource	MBS448122	81,030,119
Rabbit anti-S100	1:500	Abcam	ab868	GR3175578-1
Rabbit anti-S100b	1:5000	Synaptic Systems	287,003	1–8
Rabbit anti-GAP43	1:1000	Abcam	ab16053	GR3205552-1
Rabbit anti-synapsin-1	1:200	Cell Signaling	5297	4
Mouse anti-β-tubulin III	1:2000	Sigma	T8578	119M4757V
Goat anti-Shh	1:2000	R& D Systems	AF464	BIP1218101
Rat anti-keratin8	1:1000	DSHB	TROMA-1	5/7/2020
Rabbit anti-P2X3	1:2000	Alomone	APR-016	APR016AN0550
Rabbit anti-NF heavy	1:1000	Novus	NB300-135	216–110,516
Rabbit anti-NF light	1:5000	Novus	NB300-131	219–071,616
Chicken anti-NF heavy	1:1000	Abcam	ab4680	GR3241438-10
Rabbit anti-keratin 20	1:1000	Cell Signaling	13,063	3
**Secondary antibodies**				
Anti-rabbit Alexa Fluor 488	1:500	Invitrogen	A2120	2156521
Anti-rabbit Alexa Fluor 568	1:500	Invitrogen	A10042	2207536
Anti-mouse Alexa Fluor 568	1:500	Invitrogen	A10037	2110843
Anti-goat Alexa Fluor 568	1:500	Invitrogen	A11057	2160061
Anti-rat Alexa Fluor 568	1:500	Invitrogen	A11077	2217022
Anti-chicken Alexa Fluor 488	1:500	Invitrogen	A11039	2180688

### Neurophysiology

Mice were anesthetized via intraperitoneal injection with a ketamine/xylazine mixture (ketamine: 80–100 mg/kg; xylazine: 5–10 mg/kg), and anesthesia was maintained with ketamine (80–100 mg/kg). As described in the Methods from our previous papers (Kumari et al. 2015, 2018), mice were secured in a head holder and placed on a warming pad. The trachea was cannulated, and both hypoglossal nerves were cut to prevent tongue movements. The CT was dissected via a lateral approach, desheathed, cut centrally, and positioned on a recording electrode with a nearby reference electrode. Silicone elastomer (World Precision Instruments) was flowed into the dissected nerve cavity to maximize recording stability. The tongue was extended for stimulus access and secured by sutures through the ventral tip. Reagent grade chemicals, dissolved in distilled water, were 0.10, 0.50 M NaCl, 0.10, 0.50 M NH_4_Cl, 0.01 N HCl, 1.00 M sucrose, 0.04 M quinine HCl (QuHCl), 0.10 M citric acid, and 0.50 M L-glutamic acid monosodium salt hydrate (MSG). Stimuli at room temperature were applied to the anterior tongue for 20 s via syringe, followed by a water rinse for at least 30 s. NaCl or NH_4_Cl was applied three to four times throughout the series to monitor recording stability. The entire stimulus series was performed at least twice. Tactile stimuli consisted of light stroking of the anterior tongue three to five times with a wooden rod, applied at the beginning and end of each chemical series. Neural activity was amplified with a Grass P511 preamplifier, displayed on an oscilloscope and monitored by an audio amplifier. Amplified activity was digitized, passed through an integrator circuit, and then stored using Spike2 version 4 software (Cambridge Electronic Design). Data were quantified by measuring the height of recorded responses to chemicals above baseline at 5–10 s after stimulus application. Raw data responses for tactile stimuli were compared with vehicle and sonidegib-treated mice as in our previous studies (Donnelly et al. 2017; Kumari et al. 2017, 2018). CT recordings were made from 2 vehicle-treated and 2 sonidegib-treated mice and compared for reproducibility to recordings in previous studies (Kumari et al. 2017).

### FP and TB analysis

For each anterior half tongue, 600 μm of the mid-region of serial sagittal sections was analyzed, to exclude the median furrow or lateral tongue edges, as reported and described previously in Methods (Kumari et al. 2015, 2017). This ensured that papillae were not studied in “rounded” lateral tongue orientations or from tongue regions with no or very few papillae. In serial sections, FP and TB were characterized as follows. Typical FP/TB: The cylindrical papilla has a wide connective tissue core covered with an epithelium that includes a single apical TB; Atypical FP/TB: The papilla has lost its cylindrical shape and has a more narrow, conical form with cornified apical layers in a pointed cap. Also, the TB are smaller with fewer cells and lack a typical taste pore; Atypical FP/No TB: The highly Atypical papilla is conical in shape and lacks any collections of TB cells. For analyses, we focused on specific regions of the FP that were: the central connective tissue core; the lateral connective tissue core; the TB; the epithelium just under the TB; the apical FP epithelium; and the apical FP epithelium that is extragemmal or perigemmal, i.e., outside of the TB per se. TB were identified by *Phox2b*/RFP immunoreactions, K8 immunoreactions, DAPI staining, and/or location in FP.

For the analysis of Phox2b + distributions and Phox2b + fiber extent under the apical FP epithelium, serial sections of FP were studied in five vehicle-treated *Phox2b*-Cre*;*TdTomato mice and six sonidegib-treated *Phox2b*-Cre*;*TdTomato mice. To specifically quantify the expanse of *Phox2b* + fibers after sonidegib/LDE225 or vehicle treatment, complete FP were identified in serial sections that had full length positive fibers within the papilla core, in three of the vehicle-treated and five of the sonidegib-treated mice. The central FP section from the serial sections was then identified. In the papilla apex at the connective tissue/epithelial tissue interface, just under where the TB was located or had been located, the lateral length of the positive fibers was measured in 26 vehicle-treated and 26 sonidegib-treated FP by using the line measurement tool of the Nikon NIS Software V4.51.

Because dual labelling immunoreactions or other antibodies were conducted in *Phox2b* reporter mice, all tongues yielded data on *Phox2b* (detected with RFP immunoreactions) that was compared with specific antibody expressions. Therefore, description of the expanded *Phox2b* expression in sonidegib-treated mouse tongues was replicated in all *Phox2b* mice, in both of the half tongues for each mouse. The expansion is noted in Figs. 4, 5, 7, and 8, S1, S2, S4. For the nerve-associated antibody distributions and immunoreactions, we did not quantify immunoreaction intensity but carefully documented expression of Synapsin-1, GAP43, β-tubulin, S100 and NF-H antibodies in descriptions of FP from the anterior tongue. For describing expression patterns revealed by each antibody, we analyzed 6 to 10 FP in half-tongues from 2–4 vehicle- and 2–4 sonidegib-treated mice. We only described antibody labeling patterns in FP that could be followed in serial sections. Descriptions were based on the “central” FP section that included a TB with a pore, on the apical epithelium where TB had been located, or on the full extent of the FP connective tissue core.

### Analysis of K20 expression

Cytokeratin-20 (K20) immunoreactions were performed in cryopreserved tongues from previous experiments, taken from adult mice after 24 days of conditional deletion of smoothened in the epithelium (K5-rtTA; tetO-Cre;* Smo*^*fl/fl*^), or in the whole body (Rosa26M2rtTA/+; tetO-Cre;* Smo*^*fl/fl*^), as well as control littermates (Kumari et al. 2017). In addition, K20 immunoreactions were conducted in one *Phox2b*-Cre*;*TdTomato mouse gavaged with sonidegib and one mouse gavaged with vehicle. FP were quantified in serial sections, and categorized as Typical FP/TB, Atypical FP/TB with TB remnants, or Atypical FP/NoTB (Kumari et al. 2017).

### Experimental design and statistics

We studied 2–4 vehicle-treated and 2–4 sonidegib-treated mice for each antibody. For morphological assessment, 6 to 10 FP were evaluated in each tongue. To measure the extent of *Phox2b*/RFP expression under the apical FP epithelium (Fig. 3), we used the independent samples *t* test with Levene’s test for equality of variance to compare data between treatment groups. Statistical analyses were performed using SPSS Statistics 24 software (IBM, USA). Data displayed in figures are presented as the mean ± SE with individual data points. The threshold for statistical significance was set at *p* ≤ 0.05, and the exact *p* values are given in the figure legends.

## Results

### Chorda tympani nerve fibers from geniculate ganglion neurons that project to the anterior lingual epithelium are retained after HH pathway inhibition, along with somatosensory responses, although taste buds are eliminated

**Fig. 1:**
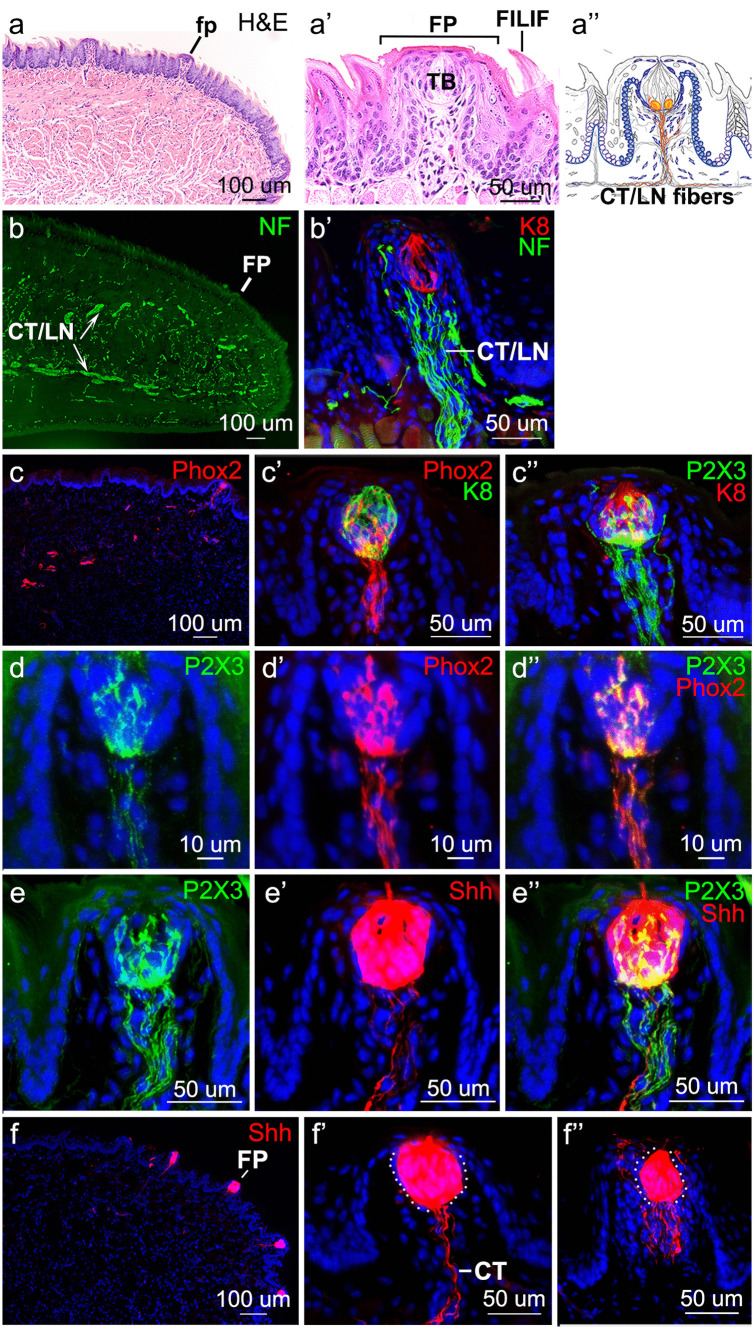
Fungiform papillae (FP) and taste buds (TB) on the anterior tongue are innervated by *Phox2b*-, P2X3*-* and *Shh*-positive chorda tympani (CT) nerve fibers. **a**,** a’** H&E staining demonstrates FP and the single apical TB and surrounding FILIF in the stratified squamous anterior tongue epithelium. **a’’** Schematic illustration of the FP displays the apical TB and innervation by CT fibers in yellow, with lingual nerve (LN) innervation (not colored) to apical and lateral FP epithelium but not to TB. Yellow cells at the TB base are locations for SHH-positive cells. Blue epithelial basal and stromal cells in the FP denote *Gli1* + cells, signaling in the Hedgehog pathway. **b**,** b’** Antibody detection of NF-H (green) in the CT/LN nerve bundles coursing through the anterior tongue and within the core of the FP **b’**. K8 + cells (red) are TB cells **b’**. **c**,** c’**,** c’’** *Phox2b* + fibers expression (seen with RFP immunoreactions in red) is shown in the anterior tongue and into the core of the FP and K8 + (green) TB cells **c**’. Antibody detection of P2X3 (green) confirms the CT location in the FP core with *Phox2b* and within the K8 + (red) TB cells **c’’**. **d**,** d’**,** d’’** Antibody detection of P2X3 colabels with the location of *Phox2b* expression in the CT and TB. **e**,**e**,**e’’** P2X3 + fibers and cells colabel with *Shh* (RFP, red, expression in *ShhCreER;* R26RFP mice) in the FP core and within TB. **f**,** f’**,** f’’** *Shh* expression is seen in all FP **f** and within CT fibers and TB cells **f’**, **f’’**. Scale bars are within each image

The FP is a cylindrical organ in the anterior tongue, covered by a stratified squamous epithelium that incorporates an apical taste bud (TB) and a connective tissue core including fibroblasts, stromal cells, blood vessels, and nerves (Fig. 1a, a’, a’’). The FP is surrounded by non-gustatory filiform (FILIF) papillae, and chorda tympani (CT) and lingual nerve (LN) fibers innervate the FP (Fig. 1a’’). Nerve bundles of the combined chorda tympani/lingual nerve (CT/LN), which can be observed with neurofilament labeling, course though the core of the tongue and take a dense trajectory to FP (Fig. 1b). In the FP, these CT/LN fibers distribute into TB (K8 +) and non-TB papilla regions within the FP (Fig. 1b’).

To distinguish the CT from LN innervation that derives from the trigeminal ganglion, we utilized the fact that the transcription factor *Phox2b* is a valid and reliable marker of oral sensory geniculate ganglion neurons that project to the tongue via CT fibers (Ohman-Gault et al. 2017). By taking advantage of *Phox2b*-Cre; TdTomato reporter mice, we are able to unambiguously visualize RFP*-*labeled fibers (from *Phox2b*-expressing neurons) that traverse the anterior tongue, ascend into FP, and terminate within TB (Fig. 1c, c’). The RFP + fibers had a similar trajectory as fibers labeled with an antibody to P2X3, which also specifically labels CT nerve projections from geniculate ganglion neurons (Ishida et al. 2009; Fig. 1c’’). To confirm that P2X3 labels the same axons that are RFP positive, we performed P2X3 and RFP co-immunolabeling in *Phox2b*-Cre; TdTomato mice. P2X3 immunolabeling marked the same projections that were RFP-positive, supporting the reliability of these two markers for oral sensory fibers that innervate FP (Fig. 1d, d’, d’’). Furthermore, there was an extensive overlap between P2X3 and RFP labeling of *Shh*-CreER expression in fibers projecting into FP (Fig. 1e, e’, e’’). The SHH ligand in the tongue epithelium is expressed only within TB cells and CT fibers in the connective tissue core (Fig. 1f, f’, f’’), as described previously (Kumari et al. 2017; Lu et al. 2018). We noted the presence of occasional P2X3 and Shh-labeled CT fibers that projected towards TB but then innervated perigemmal regions, often terminating apical to the TB (Fig. 1c’’, f’’).

**Fig. 2:**
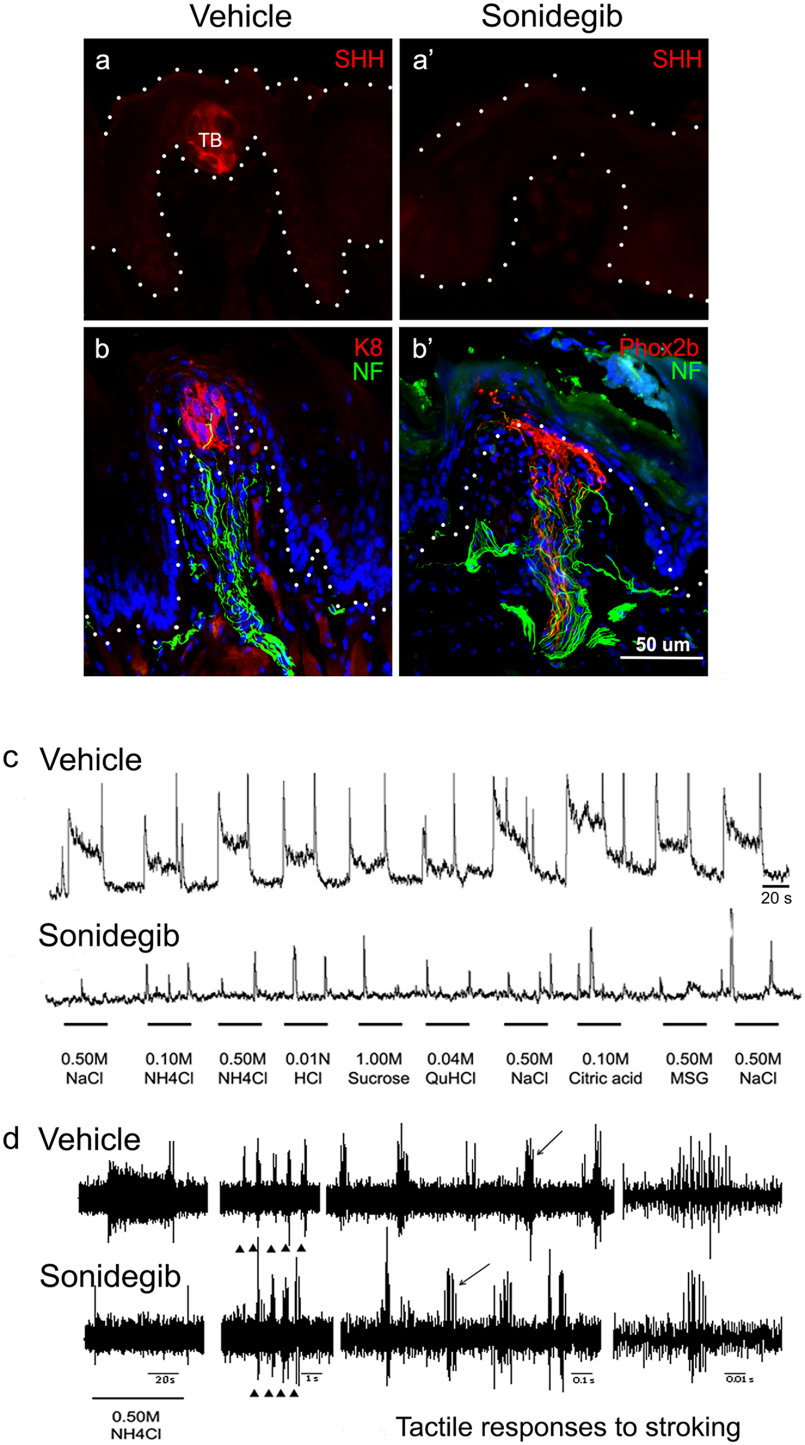
HH signaling inhibition with sonidegib treatment eliminates the TB but CT nerves remain, and these respond to tongue stroking but not to chemical taste stimuli. **a**,** a’** Antibody detection of SHH (red) in TB of vehicle-treated tongue and with HH pathway inhibition after sonidegib treatment. With TB elimination SHH expression is also lost **a’**. **b**,** b’** Antibody detection of K8 (red) in TB cells and of NF-H (green) in nerves in the FP core, in vehicle-treated tongues **b**. With sonidegib treatment TB are eliminated but the *Phox2b* + CT fibers (red) and NF-H + fibers (green) remain **b’**. Dotted lines demarcate surface and/or base of the epithelium. **c** Neurophysiological integrated recordings from the whole CT nerve in response to several chemical taste stimuli from mice treated with vehicle or sonidegib. Compared to vehicle-treated mice, HH pathway inhibition with sonidegib eliminates CT responses to a range of chemical stimuli, in association with TB loss. **d** Recordings from the whole CT nerve in response to NH_4_Cl as a taste stimulus, and from CT tactile receptors in response to stroking of the tongue. With sonidegib treatment the response to NH_4_Cl is lost, but responses to the somatosensory, stroking stimuli are retained. Triangles denote times of tongue stroke and the subsequent recordings are presented at expanded time scales to show detail of tactile responses. The last set of recordings is data in an expanded time scale from the cluster of compound action potentials indicated by the arrow in the prior set of recordings

After HH pathway inhibition with the cancer drugs sonidegib or XL139, there is an elimination of TB, resulting in an associated loss of epithelial SHH expression (Kumari et al. 2017; Lu et al. 2018; Mistretta and Kumari 2017, 2019; Fig. 2a, a’). In sonidegib treatment of *Phox2b*-Cre; TdTomato mice, NF-H + and RFP + nerve fibers remain within the FP central core even after complete TB loss (Fig. 2b, b’), confirming previous reports (Kumari et al. 2015, 2017, 2018). To evaluate neural function after HH pathway inhibition with sonidegib, we recorded from the CT nerve. In neurophysiological recordings, the TB loss is accompanied by elimination of CT responses to chemical stimulation of the tongue (Fig. 2c), but responses to lingual tactile stimuli in stroking remained (Fig. 2d), confirming prior reports (Kumari et al. 2015, 2017, 2018). The responses to each tongue stroke were sustained during stimulation and were of high frequency. There were no “off” responses, suggesting these fibers may be slowly-adapting. Thus, using the *Phox2b*-Cre; TdTomato mice in CT recordings after HH pathway inhibition enables direct determination of the somatosensory function of remaining CT fibers.

#### Oral sensory fibers from geniculate ganglion neurons that remain after HH pathway inhibition expand under the apical FP epithelium

**Fig. 3:**
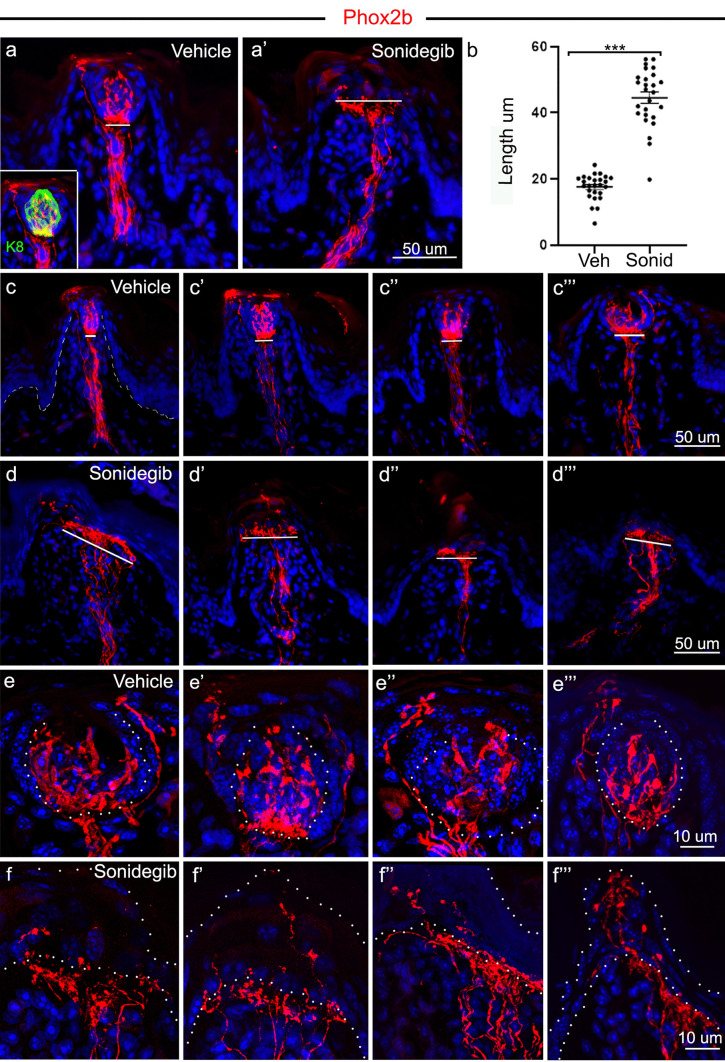
*Phox2b* expression is within the CT fibers, in TB and in perigemmal projections, and these projections are expanded under the apical FP epithelium after HH pathway inhibition. **a**,** a’** Seen with RFP immunoreactions in red, axons of *Phox2b*-expressing geniculate ganglion neurons are within CT fibers in the central FP core, within TB and in perigemmal projections within the apical FP epithelium, after vehicle or sonidegib treatment. The inset in **a** illustrates the TB labeled with K8 antibody (green). After HH pathway inhibition with sonidegib, there is an expansion of the CT fiber projections, drawn with a white line under the apical FP epithelium **a’** and compared to shorter line in vehicle-treated mice **a**. **b** Comparison of the CT fiber projection length, under the FP apical epithelium, in vehicle and sonidegib treated tongues, demonstrates a significant increase after HH pathway inhibition. Data points represent 26 vehicle- and 26 sonidegib-treated FP collected from at least 3 mice from each group. Lines are means ± standard error; circles are individual data points, ***denotes significance at *p* < 0.001; t(50) = 14.4, *p* = 0.001). **c**,** c’**,** c’’**,** c’’’** In vehicle-treated mice, the CT projection is relatively narrow and in a dense plaque just under the TB epithelium and projecting into the taste bud (demarcated with white lines in 4 different FP). **d**,** d’**,** d’’**,** d’’’** In HH pathway inhibition with sonidegib, the CT projection just under the apical FP epithelium, where TB had been located, is expanded (demarcated with white lines in 4 different FP). **e**,** e’**,** e’’**,** e’’’** In vehicle-treated mouse tongues the *Phox2b* + CT fibers are within TB (marked with dotted white lines) and in extragemmal projections that include variably clustered and spray-like endings. **f**,** f’**,** f’’**,** f’’’** In HH pathway inhibition with sonidegib, the CT projection just under the apical FP epithelium, where TB had been located, is expanded and fibers project into the FP perigemmal region around where TB had been located. The epithelial endings are complex and of varied morphology. White dots delineate the epithelium in each image. The scale bar at the end of each row **a**,** c**,** d**,** e**,** f** applies to all images within the row

To determine the nature and distribution of CT fibers that remain after HH pathway inhibition, with specificity to CT fibers from geniculate ganglion neurons, we assessed RFP + fibers in FP of *Phox2b*-Cre; TdTomato mice after oral gavage with vehicle or sonidegib. The CT extends through the center of the FP connective tissue core and into the TB, with the fibers in a compact and relatively narrow trajectory in vehicle treatment (Fig. 3a). As will be discussed in detail below, some fibers continue from this course and project into the extragemmal epithelium (Fig. 3a inset). Interestingly, RFP + CT endings, extending under the apical FP basal lamina, are expanded after HH pathway inhibition with sonidegib treatment (Fig. 3a’). Unlike the narrow, direct projection of CT fibers into the TB, when TB are lost after HH pathway inhibition, the CT fibers spread into a broader area under the apical FP epithelium where the TB had been located. The expansion is significantly larger, more than double that seen in vehicle-treated tongues (Fig. 3b). Several examples of this expansion can be seen from the comparison of RFP + fibers between vehicle-treated (Fig. 3c, c’, c’’, c’’’) and sonidegib-treated (Fig. 3d, d’, d’’, d’’’) mice.

In FP where extragemmal RFP + fibers were noted, these processes often extend all the way to the surface cells of the FP apical epithelium (Fig. 3c, c’, c’’, c’’’). While perigemmal, apical fibers in the FP have typically been considered to be from trigeminal ganglion neurons projecting via the LN, adult trigeminal ganglion neurons do not express Phox2b and, therefore, are not labeled with RFP in these studies (Donnelly et al. 2017). Interestingly, prior studies have identified extragemmal fibers from *Phox2b*-expressing geniculate ganglion neurons to be present in 43–50% of FPs (Donnelly et al. 2017; Ohman-Gault et al. 2017). Sympathetic neurons also express *Phox2b*, as well as tyrosine hydroxylase (TH), and TH-labeled fibers do project perigemmally in some FP TBs, but they do not project up to the apical surface cells of the FP (Ohman-Gault et al. 2017), indicating that the apically projecting, extragemmal RFP + fibers we observe are from CT fibers of the geniculate ganglion.

We quantified the number of FP that had apically projecting extragemmal, RFP-labeled fibers and observed that 55% of FP, out of a total of 117 papillae examined in two tongues, had such innervation, similar to other reports (Donnelly et al. 2017; Ohman-Gault et al. 2017). We further examined the peri- and extra-gemmal fibers in the FP apical epithelium to define the nature of these projections. In vehicle-treated tongues, RFP + perigemmal and extragemmal fibers and endings are diverse, including simple, thin, or branched endings, complex clusters, and thin fibers with varicosities that distribute throughout layers of the apical FP epithelium (Fig. 3e, e’, e’’, e’’’). After HH pathway inhibition, in addition to the expansion under the apical FP epithelium, there remained RFP + fibers and endings within the apical epithelium, which is now devoid of TB (Fig. 3f, f’, f’’, f’’’). Remarkably, these endings were frequently in complex clusters similar to those of vehicle-treated FP. The nature of these complex CT, extragemmal endings in the FP apical epithelium, in both vehicle and sonidegib-treated tongues, are further demonstrated in additional examples of RFP-labeled endings in Fig. S1. Overall, axons from Phox2b-expressing, oral sensory geniculate ganglion neurons project into TBs and to the FP apical epithelium, and HH-signaling inhibition that eliminates TBs results in the reorganization and expansion of these fibers at the apex of the FP epithelium/connective tissue interface, but typically with retention of the projections to the apical epithelium. This coincides with the observations that CT responses to chemical stimuli are lost after HH pathway inhibitor treatment, whereas tactile stroking responses remain (Fig. 2).

#### Evaluation of synaptic proteins and axonal remodeling in CT fibers after HH pathway inhibition

**Fig. 4:**
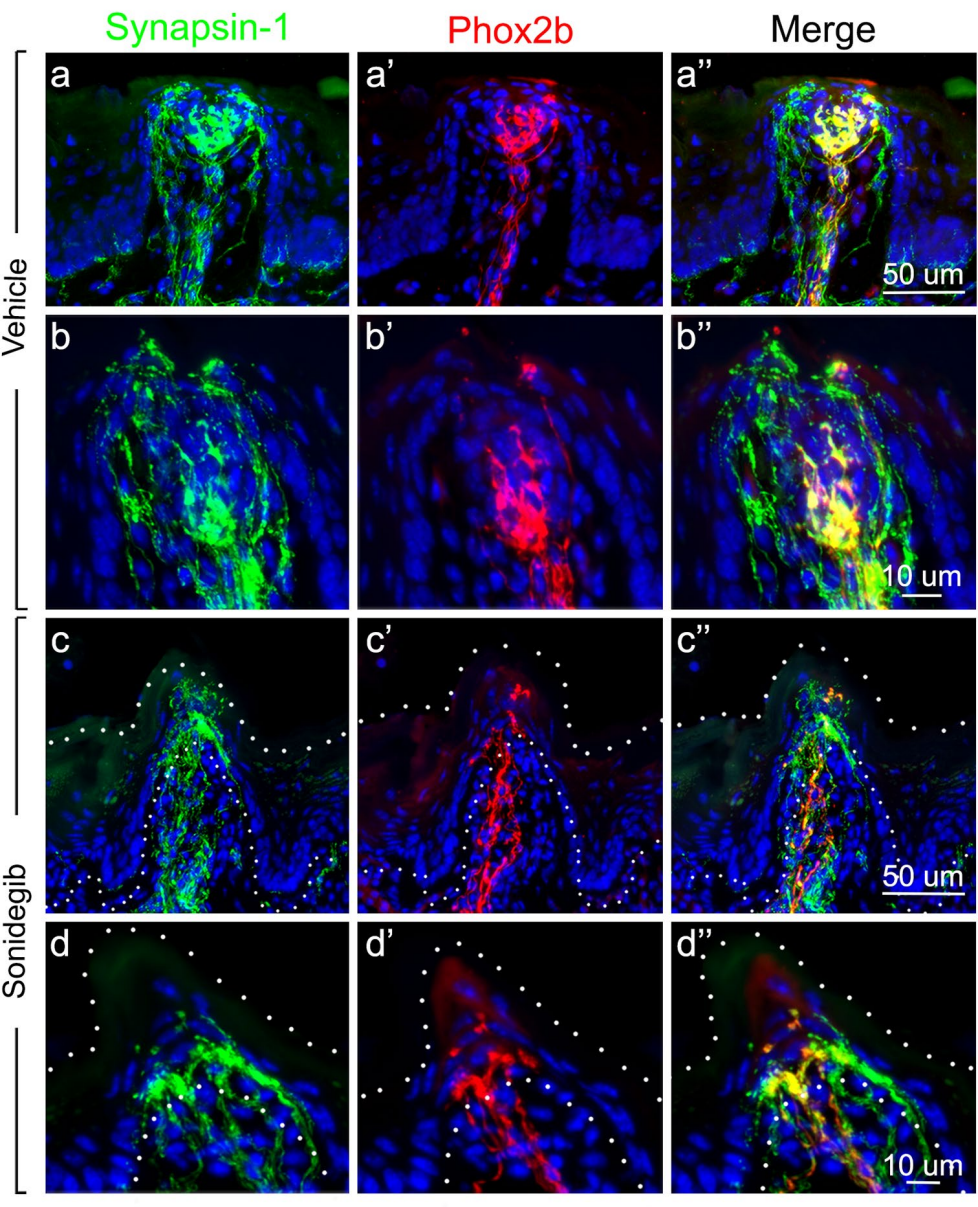
Synapsin-1 expression colabels with *Phox2b* expression in FP, TB, and within fibers that extend beyond the CT *Phox2b* projections. **a**,** a’**,** a’’** Antibody detection of synapsin-1 (green), *Phox2b* expression (seen with RFP immunoreactions in red) and merged images, in FP and TB after vehicle treatment. These images illustrate colabeling of CT fibers in the central papilla core and in TB cells. Synapsin-1 + fiber endings also extend in perigemmal epithelium and are *Phox2b* positive **a’’**. Some synapsin-1 + neural elements also extend beyond the CT in the FP core and in perigemmal endings. **b**,** b’**,** b’’** FP images at higher magnification of TB and perigemmal regions emphasize extensive synapsin-1 + endings. **c**,** c’**,** c’’** Antibody detection of synapsin-1 (green), *Phox2b* expression (seen with RFP immunoreactions in red) and merged images of FP and TB after sonidegib treatment, which illustrate the loss of TB, whereas CT innervation **c’** and synapsin-1 + fibers **c** remain. **d**,** d’**,** d’’** FP with higher magnification of TB and perigemmal regions emphasize synapsin-1 + **d** and *Phox2b* + **d’** endings. In the apical FP region just under the epithelium where TB had been located, synapsin-1 and *Phox2b* expression co-label nerve endings **d’’**. Dotted lines in rows c and d demarcate surface and base of the epithelium. The scale bar at the end of each row **a**,** b**,** c**,** d** applies to all images within the row

To determine the expression patterns of synaptic markers in the CT endings in TB and the apical FP epithelium, and how they respond to HH inhibition, we used an antibody to synapsin-1, a protein associated with synaptic vesicles, to label these fibers in vehicle-treated and sonidegib-treated mice. We observed synapsin-1 expression in fiber bundles in the FP core and in fibers in TB of vehicle-treated mice (Fig. 4a). Synapsin-1 expression overlapped extensively with the RFP labeling of *Phox2b*-Cre*;* TdTomato mice that labels CT fibers (Fig. 4a, a’, a’’). Synapsin-1 expression, however, went well beyond the RFP + CT fiber distributions in the papilla core and apical epithelium of FPs. Within the apical, extragemmal FP epithelium, RFP + fibers co-expressed synapsin-1, and there were also complex synapsin-1 + fiber extensions above TB that did not express RFP/*Phox2b* (Fig. 4b, b’, b’’). After HH pathway inhibition with sonidegib treatment, synapsin-1 remained co-expressed with RFP in CT fibers in the FP connective tissue core (Fig. 4c, c’, c’’). Notably, just under the FP apical epithelium in the region of expanded *Phox2b* + fibers, there was extensive overlap with synapsin-1, indicating that these expanded CT fibers continued to express synaptic proteins. In the FP epithelium, although TB were absent, there remained synapsin-1 + fiber endings in the FP, which take on a more conical shape after HH pathway inhibition (Fig. 4c, c’, c’’; d, d’, d’’). Synapsin-1 expression was also examined in combination with immunoreactions to β-tubulin, a general marker of neuronal microtubules. There was an extensive overlap between synapsin-1 and β-tubulin in fibers throughout the FP core and apical epithelium (Fig. S2a, a1, a2). Co-expression of synapsin-1 and β-tubulin was robust in both vehicle- and sonidegib-treated mouse tongues (Fig. S2). After HH pathway inhibition with sonidegib, the synapsin-1 + and β-tubulin + fibers under the apical FP basal lamina are expanded (Fig. S2b, b1, b2; e, e1, e2), similar to that observed with apical RFP + CT endings in Fig. 3. The data point to the maintenance of synaptic proteins even in the expanded CT nerve endings after TB elimination with sonidegib treatment.

**Fig. 5:**
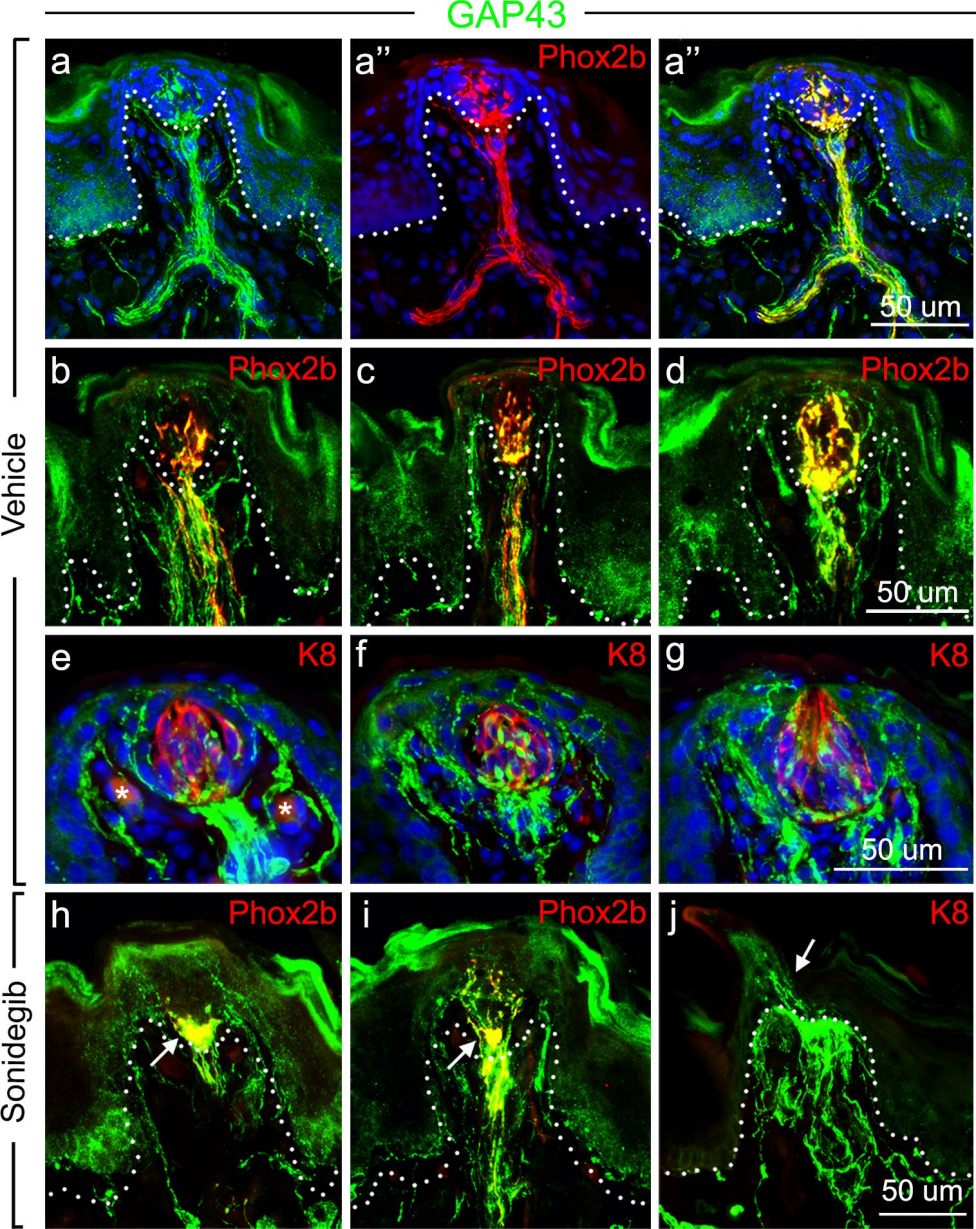
GAP43 expression colabels with *Phox2b* expression in FP and TB, but also within fibers that extend beyond the CT *Phox2b* projections. (**a, a’, a’’**) Antibody detection of GAP43 (green, a), *Phox2b* expression (seen with RFP immunoreactions in red, a’) and the merged image (a’’), in FP and TB after vehicle treatment, which illustrate colabeling within CT fibers in the papilla core and in TB cells. (**b, c, d**) Antibody detection of GAP43 (green) and *Phox2b* expression (seen with RFP immunoreactions in red) in three FP illustrate colabeling with CT fibers, and also GAP43 + fiber endings that are not *Phox2b* positive, which extend to lateral FP walls and in the perigemmal epithelium. (**e, f, g**) TB cells labeled with K8 antibody (red) in three different FP emphasize the extensive, complex GAP43 + endings in the TB and perigemmal regions. (asterisks in the stroma in ‘e’ indicate weak K8 staining intermittently observed in blood vessels). (**h, i, j**) GAP43 expression (green) with *Phox2b* (seen with RFP immunoreactions in red) (h, i) or K8 immunoreaction (j), all after sonidegib treatment. These images demonstrate that upon TB elimination during HH pathway inhibition the CT innervation remains in the papilla core and extends into the perigemmal TB region. GAP43 + and *Phox2b* + , CT innervation colabel in the apical FP plaque region just under epithelium where the TB had been located (arrow in h; i). GAP43 + fibers are throughout the FP core and into the apical epithelium (arrow in j). Dotted lines in rows a, b, and h demarcate the base of the epithelium. The scale bar at the end of each row (**a, b, e, h**) applies to all images within the row

Because HH pathway inhibition disrupts taste papilla integrity, and TB cells continually turnover in homeostasis (Mistretta and Kumari 2017, 2019), the nerves within the FP core are likely to be remodeling and have characteristics of injured/regenerating fibers. To examine this in further detail, we performed immunolabeling for growth associated protein 43 kDa (GAP43) which is associated with neuronal growth cones at the tips of elongating axons and is upregulated dramatically in peripheral axons after injury (Chong et al. 1992). GAP43, however, is also expressed in non-myelinating Schwann cells (Curtis et al. 1992). During the homeostatic, continued replacement of TB cells throughout the lifespan, CT fibers are constantly reassembling functional synaptic connections and are reported to express GAP43 normally (Zaidi et al. 2016). Consistent with this, in vehicle-treated mice, an overlapping expression of GAP43 in RFP + fibers was apparent in FP (Fig. 5a, a’, a’’). Interestingly, GAP43 + fibers also extended well beyond the central CT fiber bundle (Fig. 5b, c, d), projecting to lateral FP regions that were devoid of RFP + fibers. Within the apical FP epithelium, GAP43-expressing nerve endings were in perigemmal locations (Fig. 5e, f, g) in which they only partially overlapped with RFP. After HH pathway inhibition, in the absence of TB, GAP43 + fibers co-localized with RFP + fibers in a dense plaque under the FP apical epithelium where TB had been (Fig. 5h, i). Also, GAP43 + endings were seen in the apical FP epithelium (Fig. 5j) with no TB remaining as shown by absence of K8 expression. In summary, the GAP43 expression in vehicle- and sonidegib-treated mouse FP suggests that CT fibers are actively remodeling, both during homeostasis and after HH pathway inhibition.

#### CT fibers that traverse extragemmal regions of FP are associated with Schwann cells and these remain associated after HH pathway inhibition

**Fig. 6:**
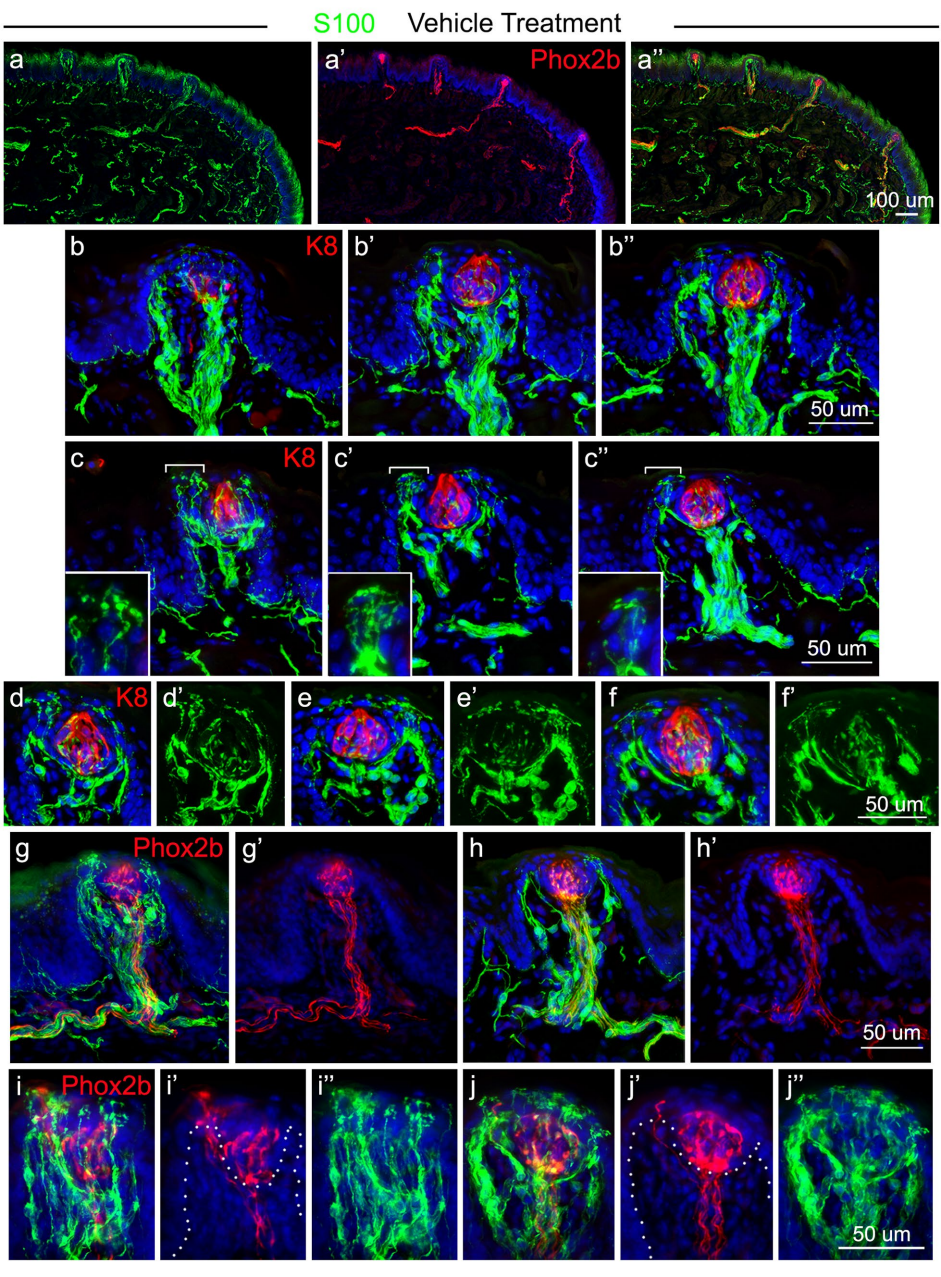
S100 expression is extensive in the FP innervation and within the perigemmal epithelium in tongues of control, vehicle-treated mice. **a**,** a’**,** a’’** Antibody detection of S100 (green, **a**), *Phox2b* (seen with RFP immunoreactions in red, **a’**) and the merged image **a’’** after vehicle treatment illustrate co-labeling with CT fibers in the anterior tongue and projecting into FP. **b**,** b’**,** b’’** Antibody detections of S100 (green) and K8 (red) label of TB cells, illustrating S100 labeled fibers in the FP core, in central and lateral projections, and in the perigemmal epithelium. **c**,** c’**,** c’’** Antibody detection of S100 and K8 (red) label of TB cells, emphasizing projections into the perigemmal epithelium. Regions with brackets are shown at higher magnification in insets for each image. **d**,** d’**,** e**,** e’**,** f**,** f’** Three TB (K8 + label) emphasize the extensive S100 + fiber endings around the TB. **g**,** g’**,** h**,** h’** Within the FP core, S100 + fibers colabel with the CT (*Phox2b/*RFP expression, red) but also extend beyond the CT to the lateral papilla walls, shown in two FP. **i**,** i’**,** i’’**,** j**,** j’**,** j’’** Antibody detection of S100 (green) and *Phox2b* (seen with RFP immunoreactions in red) in apical papilla and TB of two FP to emphasize the extensive S100 + fibers in the apical FP and perigemmal endings. Dotted lines in **i’** and **j’** demarcate the basal layer layer of the epithelium. The scale bar at the end of each row **a**,** b**,** c**,** d**,** g**,** i** applies to all images within the row

To determine whether oral sensory axons from the CT are associated with Schwann cells, we performed immunoreactions with an S100 antibody to identify myelinating and non-myelinating Schwann cells in the tongue and FP. S100 labeling was associated with nerves throughout the anterior tongue, including those that express RFP in *Phox2b*-Cre*;* TdTomato mice (Fig. 6a, a’, a’’). Within the FP, S100 + fibers are in large, discrete bundles within the papilla core traversing from the papilla base, which then split into smaller bundles that distribute to FP walls, lateral, apical, and under TB, as shown in three different papillae (Fig. 6b, b’, b’’). The bundles branch into multiple complex endings within the apical FP epithelium (Fig. 6c, c’, c’’). These endings form fine-branched clusters, with either rounded or blunt endings, and frequently have complex fibers with varicosities (Fig. 6d, d’; e, e’; f, f’). While the majority of S100 immunoproduct was extragemmal, there were occasional S100 + fibers within the TB.

The central S100 + bundles are localized with CT, RFP-expressing fibers in *Phox2b*-Cre; TdTomato mice (Fig. 6g, g’; h, h’). However, because RFP + fibers are restricted to the center of the FP core, there was no overlapping expression of RFP with S100 + fibers in laterally located trajectories. In the perigemmal epithelial region, RFP + fibers were extensively colocalized with S100, but S100 + fibers and endings also extended beyond RFP expression (Fig. 6i, i’, i’’; j, j’, j’’). In general, whereas RFP + fibers often colocalized with S100, there was not extensive colocalization in lateral and apical FP regions because of the extensive amount of S100-labeled projections. These S100 + fibers and endings that do not overlap with RFP-labeled fibers are likely to be associated with trigeminal projections from the LN. To confirm that these data faithfully represent Schwann cell elements, we used a second antibody that is specific to S100B and observed the same immunolabeling patterns in FP (Fig. S3).

**Fig. 7:**
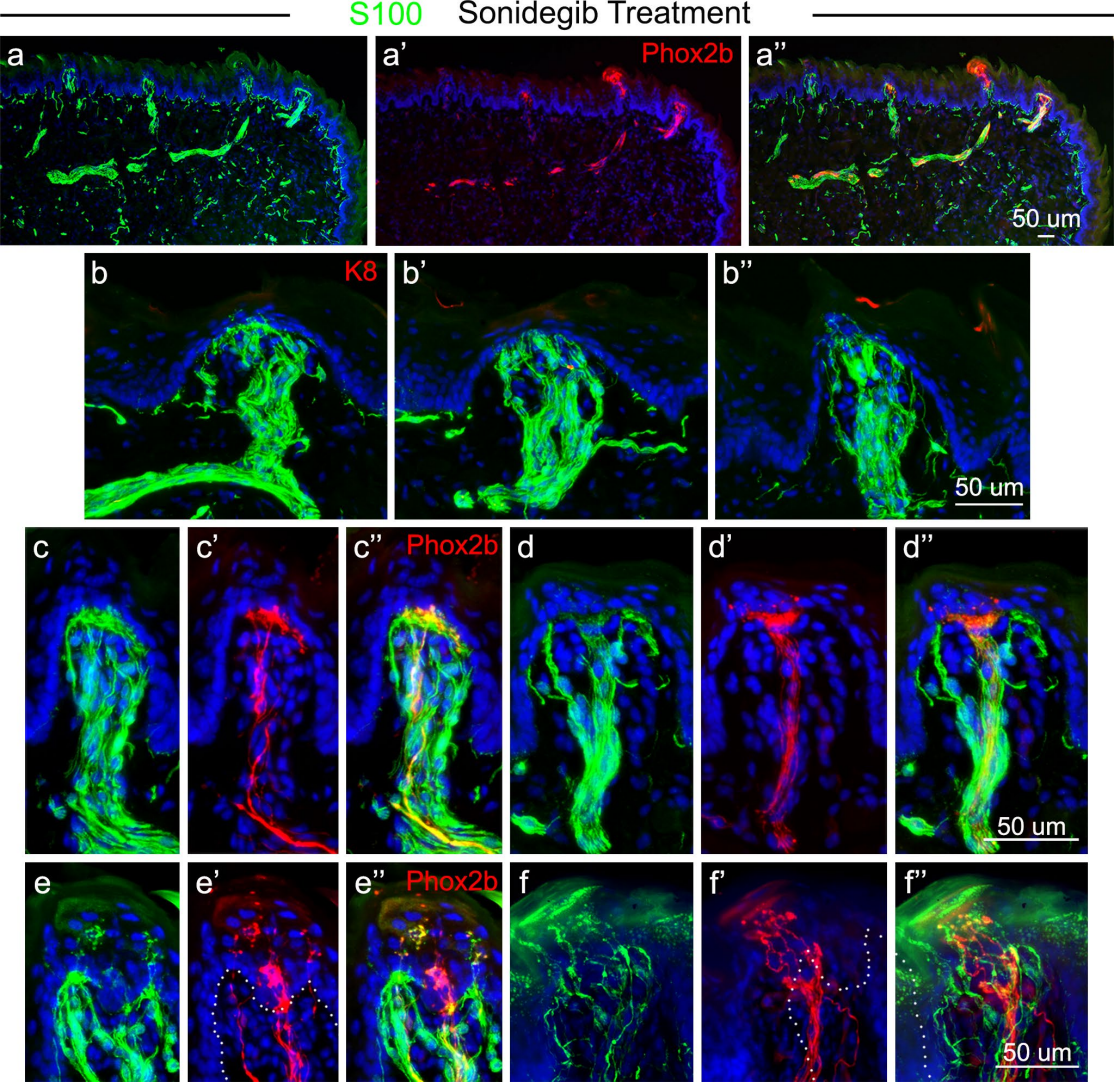
S100 expression is extensive within the FP innervation and in the perigemmal epithelium innervation in tongues of sonidegib-treated mice. **a**,** a’**,** a’’** Antibody detections of S100 (green, **a**), *Phox2b* expression (seen with RFP immunoreactions in red, **a’**) and merged image **a’’** after sonidegib treatment illustrate co-labeling of CT fibers in the anterior tongue and projecting into FP. **b**,** b’**,** b’’** Antibody detections of S100 (green) and K8 (red) label the TB cells, illustrating S100-labeled fibers in the FP core, in central and lateral projections and in the perigemmal epithelium after sonidegib treatment that eliminates TB. **c**,** c’**,** c’’**,** d**,** d’**,** d’’** Antibody detections of S100 and *Phox2b* (seen with RFP in red) demonstrate that CT fibers co-label with S100 + fibers in the FP core and in the expanded CT region under the apical FP epithelium where TB had been located. **e**,** e’**,** e’’**,** f**,** f’**,** f’’** In two different FP the S100 + fibers within the perigemmal FP region co-label with *Phox2b* expression. Dotted lines in **e’** and **f’** demarcate the basal layer of the epithelium. The scale bar at the end of each row **a**,** b**,** c**,** e** applies to all images within the row

After treatment with sonidegib, S100 expression remained throughout fibers in the anterior tongue including those that express RFP (Fig. 7a, a’, a’’). Large and discrete S100 + nerve bundles were within FP, as in vehicle-treated mice, although TB have been lost and K8 expression was thus eliminated from the epithelium (Fig. 7b, b’, b’’). An overlap of S100 expression with RFP + fibers within the central FP core was retained (Fig. 7c, c’, c’’; d, d’, d’’) in patterns similar to vehicle-treated mice. Notably, S100 expression is expanded under the FP apical epithelium, in concert with the broader distribution of RFP + fibers after HH pathway inhibition and the associated loss of TB (Fig. 7c, c’, c’’, d, d’, d’’). In the apical surface epithelium, S100 colocalizes extensively with RFP + endings, but there are more S100 + fibers in this region that are not RFP + , and overall, these S100 + endings do not appear to be significantly altered by HH pathway inhibition with sonidegib (Fig. 7e, e’, e’’; f, f’, f’’).

#### Comparing RFP expression with synapsin-1, β-tubulin, and S100 in FP

**Fig. 8:**
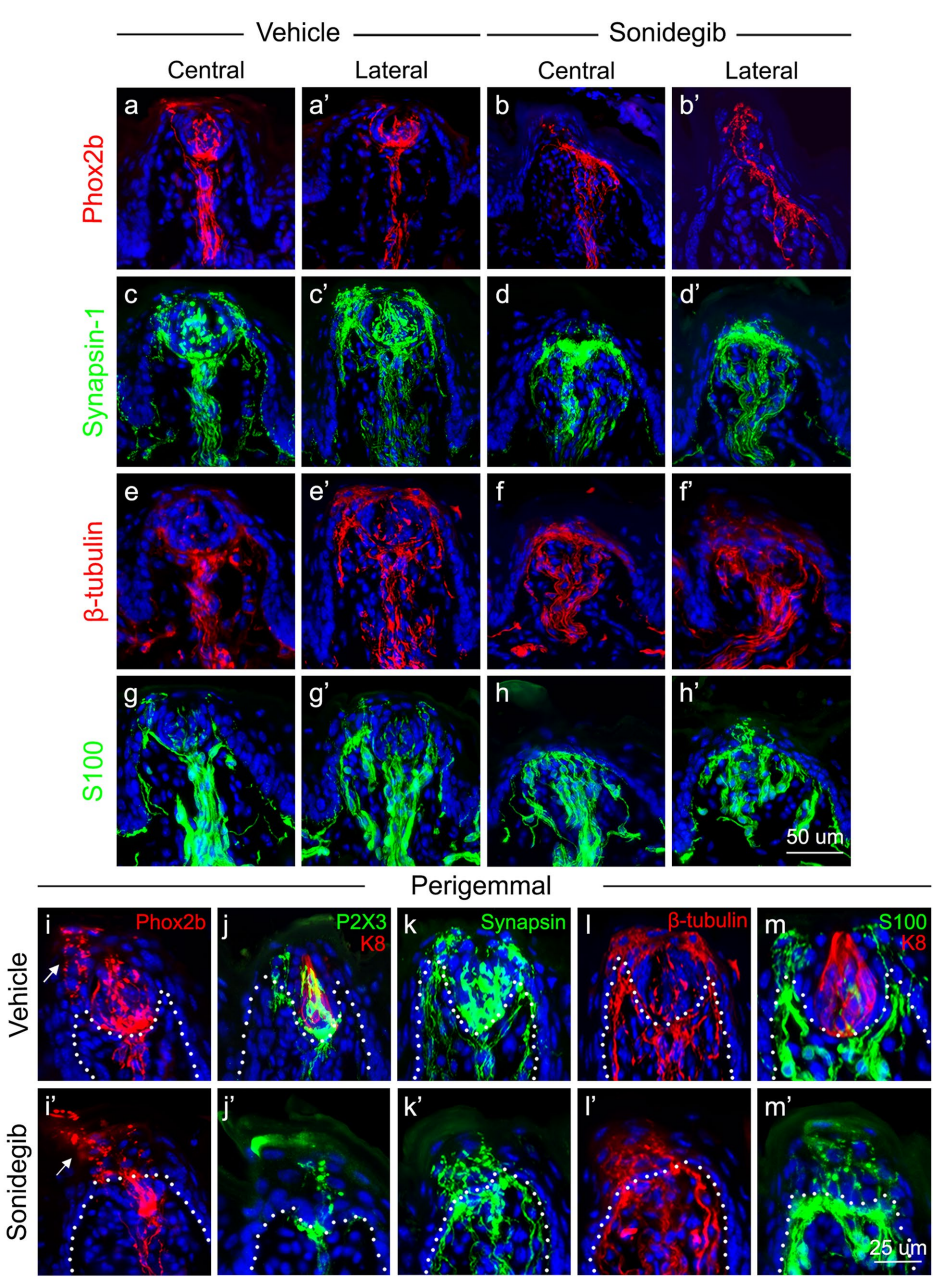
Summary of *Phox2b*, synapsin-1, β-tubulin, and S100 expression in the FP after Vehicle or Sonidegib treatment, and a summary for *Phox2b*, P2X3, synapsin-1, β-tubulin, and S100 expression in a specialized perigemmal cluster of endings. After vehicle and sonidegib treatments, expression of *Phox2b* **a**,** a’**,** b**,** b’** red, Synapsin-1 **c**,** c’**,** d**,** d’** green, β-tubulin **e**,** e’**,** f**,** f’** red, and S100 **g**,** g’**,** h**,** h’** green are compared. For each label, two views are presented: within the central FP core, and in the lateral papilla. *Phox2b* expression is within the CT fibers and TB of mice with vehicle treatment **a**, **a’** and expanded under the apical FP epithelium after HH pathway inhibition with sonidegib **b**, **b’**. In vehicle treatment, synapsin-1 **c**, **c’**, β-tubulin **e**, **e’**, and S100 **g**, **g’ **expression all overlap with the *Phox2b* + fibers, but extend beyond the CT within the FP core and perigemmal epithelium. After sonidegib treatment, all labels **d**, **d’**, **f**, **f’**, **h**, **h’** are expressed within the expanded CT region under the apical papilla epithelium. The scale bar in **h’** applies to all images within **a-h’**. After vehicle and sonidegib treatments, expression of *Phox2b* (**i, i’** red), P2X3 (green) and K8 (red) **j**,** j’**, Synapsin-1 **k**,** k’** green, β-tubulin **i**,** i’** red, and S100 **m**,** m’** green is compared to illustrate the specialized cluster of endings in the perigemmal epithelium (see area with arrow in **i**, **i’**). The dotted lines in each image demarcate the basal layer of the epithelium. The scale bar in **m’** applies to all images within **i-m’**

To summarize the data described here, we present two images for each marker from vehicle-treated mice, one through the center of the entire FP (Fig. 8a, c, e, g) and a second toward the edge of the same FP (Fig. 8a’, c’, e’, g’), illustrating the importance of FP orientation, and therefore the assessed FP area, in drawing conclusions about protein expression. In vehicle-treated mice, RFP is robustly expressed in the CT innervation in FP, in the central core fiber bundle, in the apical FP area just under the TB, and within the TB (Fig. 8a, a’). Synapsin-1, β-tubulin, and S100 are also expressed within the central CT bundle, within the TB, and within fibers that course along apical epithelium in perigemmal sites (Fig. 8c, c’, e, e’, g, g’). However, in distinction from the RFP + CT projections, fibers that express these latter three markers also project in bundles along the lateral FP walls.

After HH pathway inhibition with sonidegib treatment, the RFP-expressing CT fibers expand under the apical papilla epithelium (Fig. 8b, b’). Notably, the CT fibers do not leave the central CT bundle and turn directly out to the lateral FP walls in the mid-papilla. This suggests that the apical FP tissue is distinct from that of the lateral walls. The apical expansion of RFP-expressing CT fibers is seen also with synapsin-1, β-tubulin, and S100 immunolabeling that are colocalized with the expanse of RFP + fibers under the apical FP epithelium (Fig. 8d, d’; f, f’; h, h’).

Within the apical, perigemmal epithelium of the FP, RFP labeling of *Phox2b-*Cre; TdTomato mice revealed clusters of fiber endings (Fig. 8i, i’). These clusters, often appearing as a spray of varicosities, were clearly apparent by both RFP and P2X3 immunolabeling, demonstrating that these are CT fiber projections (Fig. 8i, j). The neuron-associated markers synapsin-1, β-tubulin, and S100 also label these projections and have varying degrees of overlap with RFP expression (Fig. 8k, l, m). Importantly, after HH pathway inhibition, we continued to observe these extragemmal fiber projections in apical FP epithelium where TB had once resided (Fig. 8i’, j’, k’, l’, m’). The maintenance of tactile, but not chemical, responses after sonidegib treatment argues that these CT fiber clusters in the apical, extragemmal FP epithelium are from Phox2b-expressing geniculate ganglion neurons that respond to tongue stroking observed in studies of somatosensory responses from the CT nerve.

#### Neurofilament expression in CT fibers is more limited, labeling a subset of oral sensory fibers

We used an antibody to neurofilament heavy (NF-H) to label large diameter, myelinated fibers in the FP and determine their representation in the CT. The NF-H + fibers overlapped to some extent with RFP + CT fibers in the core of the FP, and some NF-H + projections within the perigemmal apical FP epithelium were observed, but these were not extensive (Fig. S4a). After HH pathway inhibition with sonidegib treatment, the NF-H + fibers persisted within the FP core but were not expressed extensively in the FP apical epithelium (Fig. S4b). We observed similar expression patterns using an antibody to neurofilament light (NF-L) (Fig. S4c, d, e, f). Overall, there was more robust and extensive labeling of FP and specific CT fibers with antibodies to synapsin-1, β-tubulin, S100, and GAP43 than for neurofilaments (Figs. 5 and 8).

#### K20 as a Merkel cell marker in the FP

Merkel cells are known responders to innocuous touch in human skin and can be identified by the intermediate filament protein cytokeratin-20 (K20) as a Merkel cell marker (Bourane et al. 2009). We performed immunohistochemistry with an antibody to K20 in tongue sections from mice with conditional deletion of smoothened in the epithelium (K5-rtTA; tetO-Cre;* Smo*^*fl/fl*^), or throughout all tissues (Rosa26M2rtTA/+; tetO-Cre;* Smo*^*fl/fl*^), along with control littermates negative for *rtTA or tetO-Cre* (Kumari et al. 2017). We previously reported that conditional deletion of *Smo* in the epithelium, or in tissues globally, caused similar alterations in taste papilla and TB phenotypes as those observed after sonidegib treatment (Kumari et al. 2017).

**Fig. 9:**
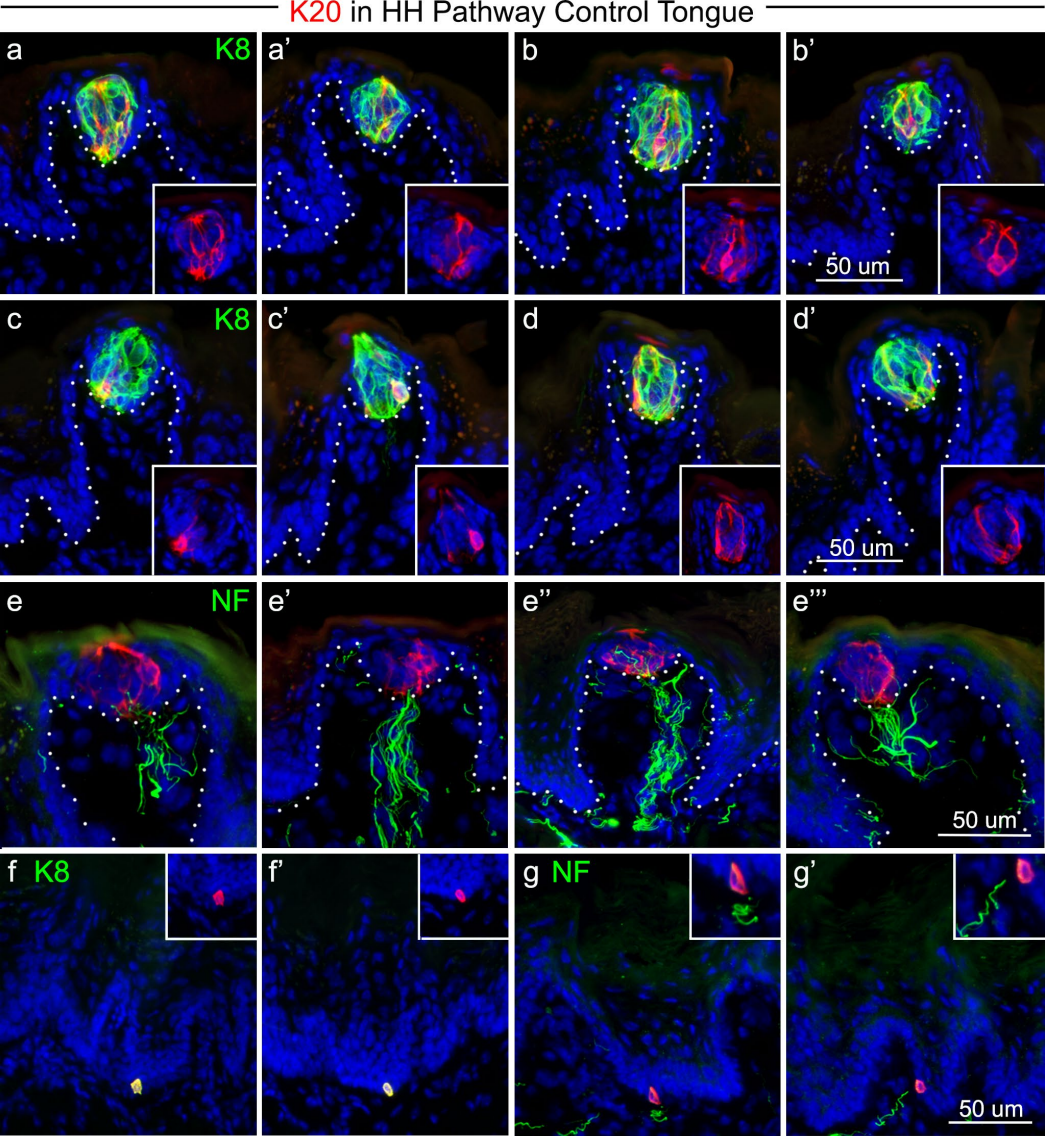
K20 + cells are in TB and in epithelial rete ridges. Antibody detection of K20 expression (red) is seen in merged images with K8 immunoreactions (green) and with NF-H immunoreactions (green), or as insets in **a** through **d’**. K20 + cells in the anterior tongue are: **a**,** a’** a subset of TB, K8 + cells; **b**,** b’** bipolar, elongate-shaped cells in TB; **c**,** c’** found in the basal cell compartment in TB; **d**,** d’** seen on the edge of TB. In four FP examples **e**,** e’**,** e’’**,** e’’’** K20 + cells are near NF-H + fiber endings (green). K20 + cells at the bottom of epithelial rete ridges also label with K8 **f**,** f’** and are located in close proximity to NF-H nerve endings **g**,** g’**. White dots in **a** through **e’’’** demarcate the basal cell layer of the FP epithelium. Data presented in this figure was obtained from *Smo* rtTA or tetO-cre littermate control animals. The scale bar at the end of each row **b’**,** d’**,** e’’’**,** g’** applies to all images within the row

Because Merkel cells have not been described in detail in the mouse tongue, we first catalogued the expression pattern of K20 in the lingual epithelium of control tongues. We found that K20 + cells were a subset of K8 + TB cells (Fig. 9a, a’) that had elongate, bipolar shapes within TB (Fig. 9b, b’). K20 + cells were frequently located as basal cells within TB (Fig. 9c, c’) and were often observed on the edge of TBs (Fig. 9d, d’). In addition, the K20 + cells within TB were in close proximity to NF-H + nerve fibers, in varying configurations (Fig. 9e, e’, e’’, e’’’). Outside of the FP and TB, K20 + cells were identified as solitary cells in the rete ridges of FILIF epithelium (Fig. 9 f, f’) and were again present in close proximity to with NF-H + fibers (Fig. 9g, g’).

**Fig. 10:**
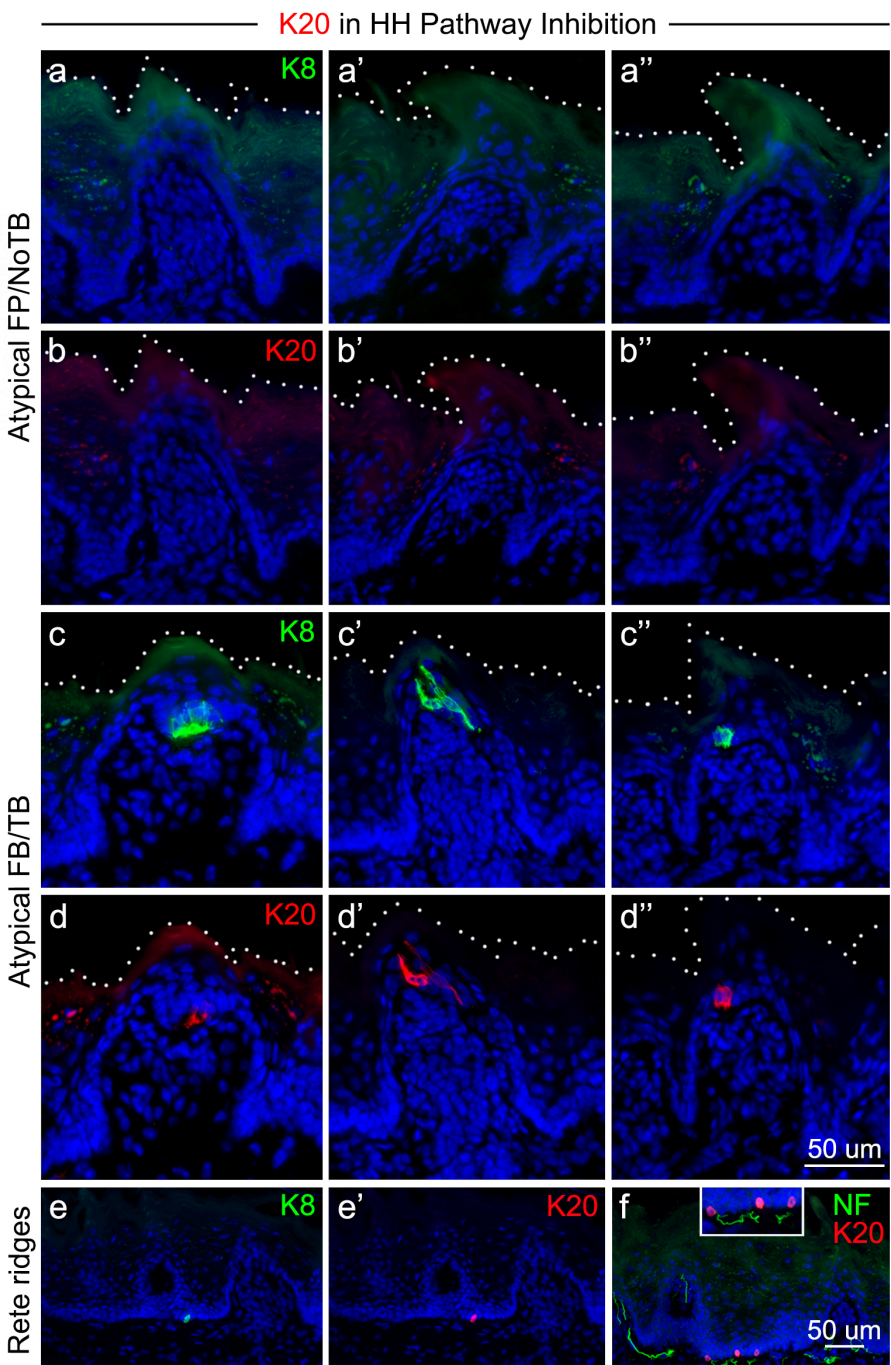
K20 + cells are lost in FP upon TB elimination after HH pathway inhibition, but remain in the epithelial rete ridges. Antibody detection of K20 (red) and K8 (green) in FP and rete ridges after HH pathway inhibition in K5-rtTA;tetO-Cre;*Smo*^fl/fl^ mice. **a**,** a’**,** a’’** and **b**,** b’**,** b’’** In Atypical FP with No TB, the K8 + TB cells are eliminated, along with K20 + cells. **c**,** c’**,** c’’’** and **d**,** d’**,** d’’’** In Atypical FP with TB remnants, some K8 + cells remain and K20 + cells also remain in these remnants. White dots in **a** through **d’’** demarcate the surface of the FP and epithelium. **e**,** e’** In epithelial rete ridges K8 + /K20 + solitary cells are retained after HH pathway inhibition. **f** The K20 + cells in epithelial ridges associate with NF-H nerve endings (and shown at higher power in inset). The scale bar at the end of row **d’’** applies to all images from a through **d’’**. The scale bar in **f** applies to images in **e**, **e’**, and **f**

When K8 + cells, or TB, were absent from FP after HH pathway inhibition with *Smo* deletion, there were also no K20 + cells in the category Atypical FP/No TB, which are atypical FP that have no remaining TB (Fig. 10a, a’, a’’; b, b’, b’’). Thus, these K20 + cells cannot explain the continued presence of CT responses to stroking following HH pathway inhibition (Fig. 2). After HH signaling inhibition, some FP retain a remnant TB containing a few K8 + cells that remain within a misshapen FP (Atypical FP/TB category; Fig. 10c, c’, c’’). We found that within these TB, there were K20 + cells (Fig. 10d, d’, d’’), some of which appeared to co-express K8 and K20. Therefore, the continued presence of K20 expression within these remnant TB correlates directly with the residual presence of K8 + TB cells. Notably, the solitary K20 + cells within the rete ridges of FILIF remain after HH pathway inhibition (Fig. 10e, e’), co-express K8, and remain associated with NFH (Fig. 10f, f inset).

## Discussion

The FP is a complex organ on the anterior tongue for lingual taste, tactile, and temperature sensations, all mediated by the CT nerve from neurons in the geniculate ganglion (Mistretta and Bradley 2021). The LN, comprised of projections from somatosensory neurons of the trigeminal ganglion, also innervates FP and also responds to lingual touch and temperature, but does not innervate TB and therefore does not respond to typical taste stimuli or chemicals at moderate concentrations (Green 2012). Given the relatively small size of FP, in conjunction with the extensive innervation by CT and LN afferents, distinguishing the patterning and spatial organization of these fibers has proven difficult. We utilized a reporter mouse based on the transcription factor *Phox2b* to specifically label the CT nerve (Ohman-Gault et al. 2017) and identify the trajectories and morphologic characteristics of the CT fibers in the FP during homeostasis, as well as after HH pathway inhibition that eliminates TB. Importantly, after sonidegib treatment, the CT nerve remains in FP and does not respond to chemical stimuli, but continues to respond to tactile and cold stimuli, demonstrating that TB are not required (Kumari et al. 2015, 2017, 2018; Lu et al. 2018). The RFP-labeled CT fibers, both before and after sonidegib treatment, were co-labeled for synapsin-1, β-tubulin, S100, and NF; co-labeling with GAP43 suggested the fibers are constantly remodeling, not just after HH pathway inhibition.

Interestingly, in alignment with previous reports (Donnelly et al. 2017; Ohman-Gault et al. 2017), we found that at least half of all FP bear CT fibers that remain outside of TB, project to the perigemmal epithelium of the FP, and have complex, branched endings that frequently contain varicosities and terminate with bulb-like expansions. These complex CT fibers in the epithelium were associated with S100 labeling, suggesting there may be end organs within these structures. Importantly, structures with this morphology were apparent after sonidegib treatment, implying that these terminals remain largely unchanged after HH pathway inhibition. Somatosensory neurons generally require end organs to transduce sensory stimuli, including “free nerve ending” nociceptors that require specialized Schwann cells (Abdo et al. 2019). Overall, the complex nerve endings apical and perigemmal to TB in the FP epithelium, and in a more disbursed distribution just under the apical FP epithelium, are the most plausible nerve endings that underlie somatosensation of the CT after HH pathway inhibition (Fig. S5).

### Neurobiological properties of mechanoreceptive chorda tympani afferents in the fungiform papilla

When considering mechanosensation from geniculate ganglion neurons that project via the CT to FP, an important question is how to define this/these population(s) molecularly and physiologically. Based on recent studies employing single-cell RNA-seq of geniculate ganglion neurons, 1–2 subpopulations have molecular signatures of Phox2b + mechanoreceptors (Dvoryanchikov et al. 2017; Zhang et al. 2019). We recently identified a subpopulation of Phox2b + geniculate ganglion neurons that express Ret, the GDNF receptor, in adult mice that are mechanosensory and not chemosensory (Donnelly et al. 2017), which is presumably the recently described T2 subpopulation identified by RNAseq that expresses Ret (Dvoryanchikov et al. 2017).

In regard to their physiologic properties, the CT mechanical responses are sustained during the stroking stimulus, have no off responses, and thus, are not rapidly adapting (Fig. 2; Kumari et al. 2015, 2017, 2018). In extracellular single-cell recordings of the CT soma in the geniculate ganglion, these neurons have a high action potential frequency, respond robustly to stroking with a sustained discharge, and respond poorly to direct pressure on FP (Yokota and Bradley 2017). Most of what is known about mechanoreceptors is based on studies of dorsal root ganglion (DRG) neurons, which have been categorized using a combination of molecular characteristics, conduction velocities, sensitivity to different types of tactile stimulation, the morphology of their end organs, and physiological properties (Zimmerman et al. 2014). A comparison to DRG mechanoreceptors leads to the conclusion that CT-projecting mechanoreceptors do not fit well into any known DRG category. These neurons share some similarities with low-threshold mechanoreceptors (LTMRs) that express Ret, respond to stroking, and have small receptive fields, although these DRG neurons have lanceolate endings around individual hair follicles (Li et al. 2011). The CT oral mechanoreceptors are also similar to Aβ field LTMRs with respect to their high sensitivity to gentle stroking with a fine brush and low sensitivity to direct pressure (Bai et al. 2015; Yokota and Bradley 2017). However, the Aβ field LTMRs have very large receptive fields, whereas oral mechanoreceptors have smaller receptive fields and are able to respond to stroking of individual FP.

It is not surprising that mechanosensory neurons in the oral cavity differ from touch-sensitive neurons that innervate skin given the unique morphological and physiological characteristics of the mouth and the tongue in particular. Responses to gentle tactile stroking might derive from specialized receptor organs, complex afferent terminal endings, or non-neuronal keratinocytes and Schwann cells (Mistretta and Bradley 2021), as in epidermal mechanotransduction (Moehring et al. 2018; Nakatani et al. 2015).

### Chorda tympani fiber endings in the fungiform papilla

In the current study, we have paid particular attention to the apical, perigemmal epithelium of the FP where CT fibers display a unique configuration of clustered fiber endings. These perigemmal fibers and endings adopt various forms that project throughout the apical epithelial layers, including simple and branched endings, thin fibers with varicosities, often with bulbous endings, and the entire complex is typically organized into a collective “spray” of endings. Similar perigemmal clusters were seen in FP in Brainbow mice and were attributed to LN projections (Zaidi et al. 2016). The comparable nerve endings we observed here using *Phox2b*-Cre; TdTomato reporter mice are unlikely to have a contribution from the LN because there are only a few *Phox2b* + neurons in the entire trigeminal ganglion, too few to account for the extragemmal TB innervation (Donnelly et al. 2017; Ohman-Gault et al. 2017). While sympathetic neurons of the superior cervical ganglion express *Phox2b* and innervate arteries of the tongue, tyrosine hydroxylase (TH) labeling of *Phox2b*-expressing nerves revealed that TH-labeled fibers, although occasionally observed in FP, do not extend into the apical epithelium (Ohman-Gault et al. 2017). Recently it was reported that extragemmal axon clusters that are labeled with β-tubulin also express *Piezo2*, an ion channel associated with mechanosensation (Moayedi et al. 2018). Interestingly, the *Piezo2* expression was seen in extragemmal clusters near the epithelium only within FP and in no other locations in the tongue (Moayedi et al. 2018). It is possible that CT fibers may respond, after HH pathway inhibition, to tactile and temperature stimuli through keratinocyte activation. Skin keratinocytes, for example, have been shown to mediate tactile responses via released ATP that signals via P2X4 receptors on sensory neurons (Moehring et al. 2018). A similar mechanism is possible by ATP released from FP keratinocytes, signaling via P2X or P2Y receptors on CT endings, although deletion of P2X2 and P2X3 receptors did not alter tactile responses in CT (Finger et al. 2005).

The tongue is more sensitive than even the fingertip in two point discrimination and in discriminating food texture (Aktar et al. 2015). Of tongue regions, the tip is the most highly innervated (Marlow et al. 1965), in direct relation to the exquisite lingual sensitivity to touch which had been generally attributed to LN innervation (Trulsson and Essick 1997). However, we have shown here and have proposed (Mistretta and Bradley 2021) that in the multimodal FP the CT nerve is a principal participant in both chemosensory and somatosensory sensitivity of the anterior tongue. Indeed, highly complex FP innervation and multimodal responses of the CT fibers raise intriguing questions about CT responses to tactile and cold stimuli in exploration and palatability of food (Mistretta and Bradley 2021) and in affective touch responses to appetitive textures.

### K20 + cells in the tongue

In human autopsy material from the oral cavity, Merkel cells, identified as K20 + , were reported in the mandibular gingiva, hard palate, and buccal mucosa (Barrett et al. 2000). All of these oral K20 + cells were also K8 + and K18 + . The only other oral location for K20 expression was in TB, and it was concluded that K20 is a specific marker of TB and Merkel cells in human oral mucosa. Recently, others have reported K20 + cells in the human hard palate and in FP taste buds (Moayedi et al. 2021). In several non-human mammals, however, no K20 + cells were found in any oral epithelium, even though in that study K20 + cells were identified in the luminal epithelium of colon (Barrett et al. 2000). In mouse tongue we observed K20 + cells in TB and in rete ridges of the lingual epithelium, which were co-labeled with K8. When TB are lost after HH signaling inhibition, K20 + cells are also lost in FP. Given that the continued presence of K20 + cells paralled that of K8 + TB remnants, and our previous study which demonstrated that fewer than half of all FP retain TB remnants after sonidegib treatment (Mistretta and Kumari 2017), it seems unlikely that the residual K20 + cells could be responsible for the robust CT responses to tactile stimuli which remain after HH pathway inhibition. Further, using K20 as a marker, we did not observe large numbers or clusters of putative Merkel cells in the tongue epithelium. This reinforces the likelihood that fibers from Phox2b + neurons, with their terminals in the FP perigemmal epithelium and just under the TB-bearing FP epithelium, are the most likely elements responding to tactile stimulation in CT recordings. Overall, these data suggest that if indeed these CT fibers respond to tactile stimuli through an as-of-yet undiscovered sensory end organ present in the lingual epithelium, it is unlikely to be chiefly mediated by K20 + Merkel cells.

In addition, we found no evidence for a Merkel cell/neurite complex in the FP that equates with those found in skin. The keratinocytes that surround the TB are specialized as Gli1 + cells, and lineage tracing experiments demonstrate that these are precursors of TB cells (Liu et al. 2013; Mistretta and Kumari 2017, 2019). The perigemmal Phox2b + fibers and other specialized fibers, that remain after TB are eliminated in HH pathway inhibition, could interact with these specialized keratinocytes. Future studies are needed to better describe the nature of CT fiber endings and associated end organs that respond to taste, mechanical and cold stimuli (Mistretta and Bradley 2021).

### The taste bud as a central organizer of afferent nerve fibers in the fungiform papilla

After HH pathway inhibition, we have shown that CT fiber bundles that projected into TB expand their innervation pattern under the FP apical epithelium, revising and redirecting the fasciculated projection to the central FP where TB normally reside. These fibers project outward into a flat disc-shaped bundle which doubles in size, indicating that the TB is critical in directing the organization of CT fibers. Notably, these fibers do not expand perpendicularly, out to the middle lateral walls of the FP. These data suggest that there are inhibitory axon guidance cues in FP limiting their exploration after TB loss. Alternatively, attractive guidance cues may keep these fibers concentrated in the central core of the FP. Interestingly, these axons do not appear to degenerate or retract from FPs, and Phox2b + oral sensory neurons of the geniculate ganglion do not degenerate or die after HH pathway inhibition (Kumari et al. 2015). This implies that the FP itself, after the loss of TB, is sufficient to support these CT fibers and their associated neurons (Mistretta and Kumari 2019). Almost half of all Phox2b + geniculate neurons in the adult require BDNF produced by TBs for their trophic maintenance (Tang et al. 2017), suggesting that either other cells in the FP are capable of providing BDNF to these fibers after HH pathway inhibition, or that other neurotrophic factors provide sufficient support. The maintenance of CT fibers within the FP after the loss of TB has important implications for regeneration, namely that the continued presence of these fibers within the vicinity of regenerating TB aids in the reinnervation of these newly emerging TB. The continued presence of these fibers is also critical for TB regeneration by providing growth factors, such as SHH (Ermilov et al. 2016; Kumari et al. 2017; Lu et al. 2018; Mistretta and Kumari 2019). While the regenerative capacity of the peripheral taste system is substantial, our molecular understanding of this process, and how it differs among fiber types and sensory modalities, is still rudimentary.

### Summary

The FP is a polymodal organ on the anterior tongue that mediates initial tongue responses to taste, touch, and temperature via the CT (Kumari et al. 2017; Mistretta and Bradley 2021; Mistretta and Kumari 2019). In this study, we investigated the fiber endings within the FP, TB, and extragemmal locations to emphasize the innervation complexities of this lingual organ. By imaging identified CT fibers in a HH pathway inhibition paradigm that eliminates the TB, we have begun to discern fibers and terminals that respond to lingual mechanosensory stroking as compared to those fibers that respond to chemical stimuli. Future experiments to identify the panoply of CT/geniculate ganglion neuron subtypes in tongue sensation, using a combination of molecular, morphological, and functional approaches, will be key to understanding multimodal tongue sensation.

## Supplementary Information

Below is the link to the electronic supplementary material.

Supplementary file1 (PDF 23 MB)

## Data Availability

The data sets generated or analyzed are available from the corresponding authors upon reasonable request.
