# Correction to: Probing the multimodal fungiform papilla: complex peripheral nerve endings of chorda tympani taste and mechanosensitive fibers before and after Hedgehog pathway inhibition

**DOI:** 10.1007/s00441-022-03642-9

**Published:** 2022-06-14

**Authors:** Christopher R. Donnelly, Archana Kumari, Libo Li, Iva Vesela, Robert M. Bradley, Charlotte M. Mistretta, Brian A. Pierchala

**Affiliations:** 1 Department of Biologic and Materials Sciences, University of Michigan School of Dentistry, Ann Arbor, MI USA; 2 Center for Translational Pain Medicine, Department of Anesthesiology, Duke University Medical Center, Durham, NC USA; 3 Duke Cancer Institute, Duke University Medical Center, Durham, NC USA; 4 Rowan University School of Osteopathic Medicine, Stratford, NJ USA; 5 Department of Anatomy, Cell Biology & Physiology, Stark Neurosciences Research Institute, Indiana University School of Medicine, Indianapolis, IN USA

## Correction to: Cell and Tissue Research https://doi.org/10.1007/s00441-021-03561-1

The authors regret that the supplementary files captured in original article are incomplete.

The original article has been corrected.

