# Supplementary material for: Probing the multimodal fungiform papilla: complex peripheral nerve endings of chorda tympani taste and mechanosensitive fibers before and after Hedgehog pathway inhibition
Source: Cell Tissue Res. 2021 Dec 3;387(2):225–47. doi: 10.1007/s00441-021-03561-1 (PMC8821500; doi:10.1007/s00441-021-03561-1)
Supplement: Supplementary file 1 — Supplementary file1 (PDF 23 MB) [file 441_2021_3561_MOESM1_ESM.pdf]

Supplementary Information

Probing the multimodal fungiform papilla: complex peripheral nerve endings of chorda tympani taste and mechanosensitive fibers before and after Hedgehog pathway inhibition

Authors: Christopher R. Donnelly[1,2,3,6], Archana Kumari[1,4,6] Libo Li[1,7], Iva Vesela[1,7], Robert M. Bradley[1], Charlotte M. Mistretta[1,8,*], Brian A. Pierchala[1,5,8,*]

[1] Department of Biologic and Materials Sciences, University of Michigan School of Dentistry, Ann Arbor, Michigan, USA.

[2] Center for Translational Pain Medicine, Department of Anesthesiology, Duke University Medical Center, Durham, North Carolina, USA.

[3] Duke Cancer Institute, Duke University Medical Center, Durham, North Carolina, USA.

[4] Rowan University School of Osteopathic Medicine, Stratford, New Jersey, USA.

[5] Department of Anatomy, Cell Biology & Physiology, Stark Neurosciences Research Institute, Indiana University School of Medicine, Indianapolis, IN, USA.

[6] These authors contributed equally and share first authorship.

[7] These authors contributed equally.

[8] These authors contributed equally and share senior authorship.

* Please address correspondence to: brpierch@iu.edu; chmist@umich.edu

Supplementary Figure Legends

Fig S1. *Phox2b* expression is extensive in the FP perigemmal epithelium

(**a, b, c, d**) *Phox2b* expression (seen with RFP immunoreactions in red) is illustrated in four FP after vehicle treatment and (**e, f, g, h**) four FP after sonidegib treatment. The images illustrate extensive perigemmal chorda tympani projections in the apical FP (arrows in all images). The scale bar in **h** applies to all images.

Fig S2. Synapsin-1+ and β-tubulin+ fibers overlap in the FP

Antibody detection of synapsin-1 (green) and β-tubulin (red) expression in vehicle-treated (**a, a1, a2**) and sonidegib-treated (**rows b, c, d, e**; four different FP) mice demonstrate, in merged images, the extensive colabeling throughout fibers in the FP. The scale bar in **e2** applies to all images.

Fig S3. S100B-specific antibody labeling is similar to that for S100

Antibody detection of S100B (**a** red) in wild type mice overlaps with, and extends beyond, NF-H (**a1** green) expression (**a2** merged). The S100B expression (red) distributes throughout innervation in the central FP core and to lateral papilla walls (**b, c**), and also courses into apical perigemmal epithelium (**d**). Examples of three other FP using antibody detection of K8 (green) and S100B (red) further illustrate the distribution of S100B in fibers within the FP and in the apical perigemmal epithelium (**e, f, g**). Shown with K8 immunoreaction (green) for TB cells, S100B expression is complex within the perigemmal epithelium, seen in four FP (**h, i, j, k**). Overall, the expression of S100B is directly comparable to that for the S100 antibody. The scale bar in **k** applies to all images.

Fig S4. NF Heavy (NF-H) and Light (NF-L) expression co-label with *Phox2b* in FP, and are also located within fibers that extend beyond the *Phox2b* projections

Phox2b expression (seen with RFP immunoreactions in red) and antibody detection of NF-H (**a, b** green) and NF-L (**c-h** green) in FP and TB after vehicle treatment (**a, c, e, g**) and sonidegib treatment (**b, d, f, h**). These images illustrate colabeling with chorda tympani fibers in the central papilla core, along with very limited NF fibers extending beyond the chorda tympani in the apical perigemmal FP epithelium. The scale bar in **h** applies to all images.

Fig S5. Diagram of chorda tympani fiber remodeling after Hedgehog pathway inhibition

The image on the left depicts a mouse FP during homeostasis. Chorda tympani fibers (blue) ascend through the papilla core and most enter the TB (brown) while some fibers remain extragemmal (pink) and project up to the apical epithelium and have complex terminations. Green cells represent S100-expressing Schwann cells. After Hedgehog pathway inhibition (right image) the TB is lost (dotted line) and intragemmal fibers remodel, collapsing into a broad band of fibers. The extragemmal fibers remain close to the apical epithelium even though remodeling of the papilla has occurred. Chorda tympani responses to chemical stimuli are lost (blue fibers) while responses to mechanical stimuli are retained, presumably from the extragemmal (pink) fibers, although tactile responses may emanate from remodeled intragemmal fibers as well.

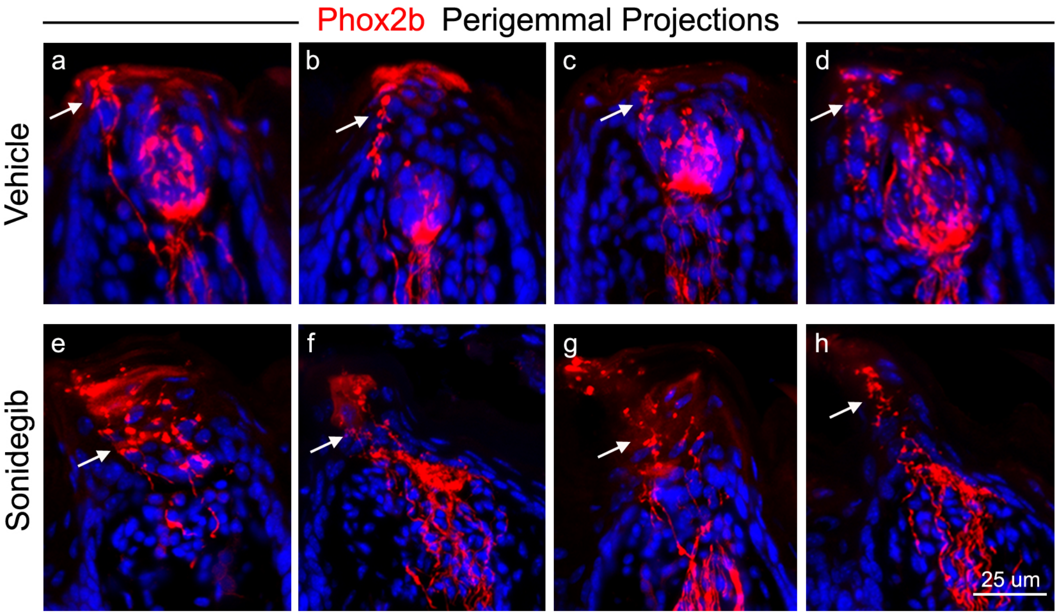

Phox2b Perigemmal Projections
Vehicle
Sonidegib
a
b
c
d
e
f
g
h
25 um

Figure S1

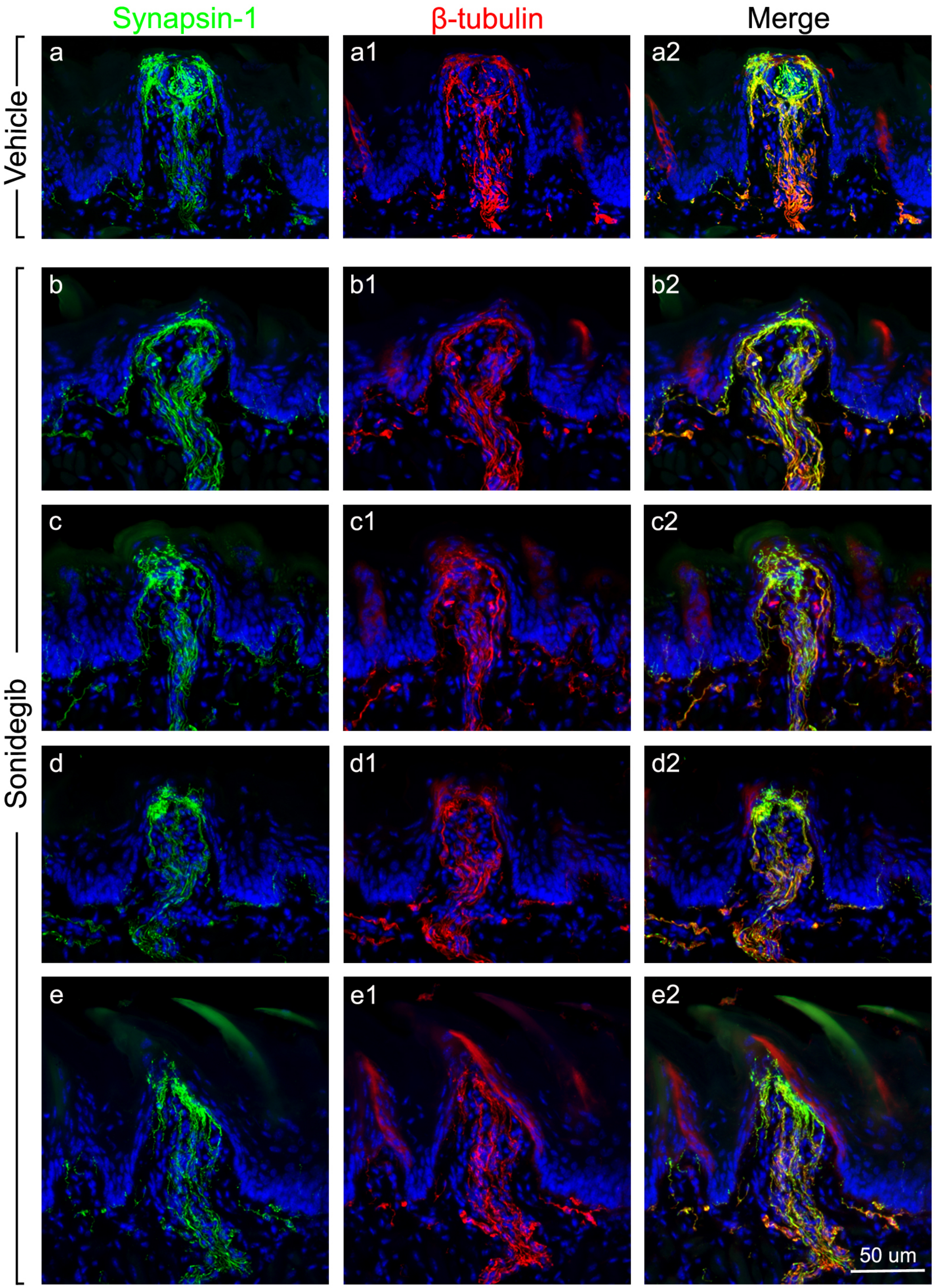
Synapsin-1
β-tubulin
Merge
Vehicle
Sonidegib
a
a1
a2
b
b1
b2
c
c1
c2
d
d1
d2
e
e1
e2
50 um

Figure S2

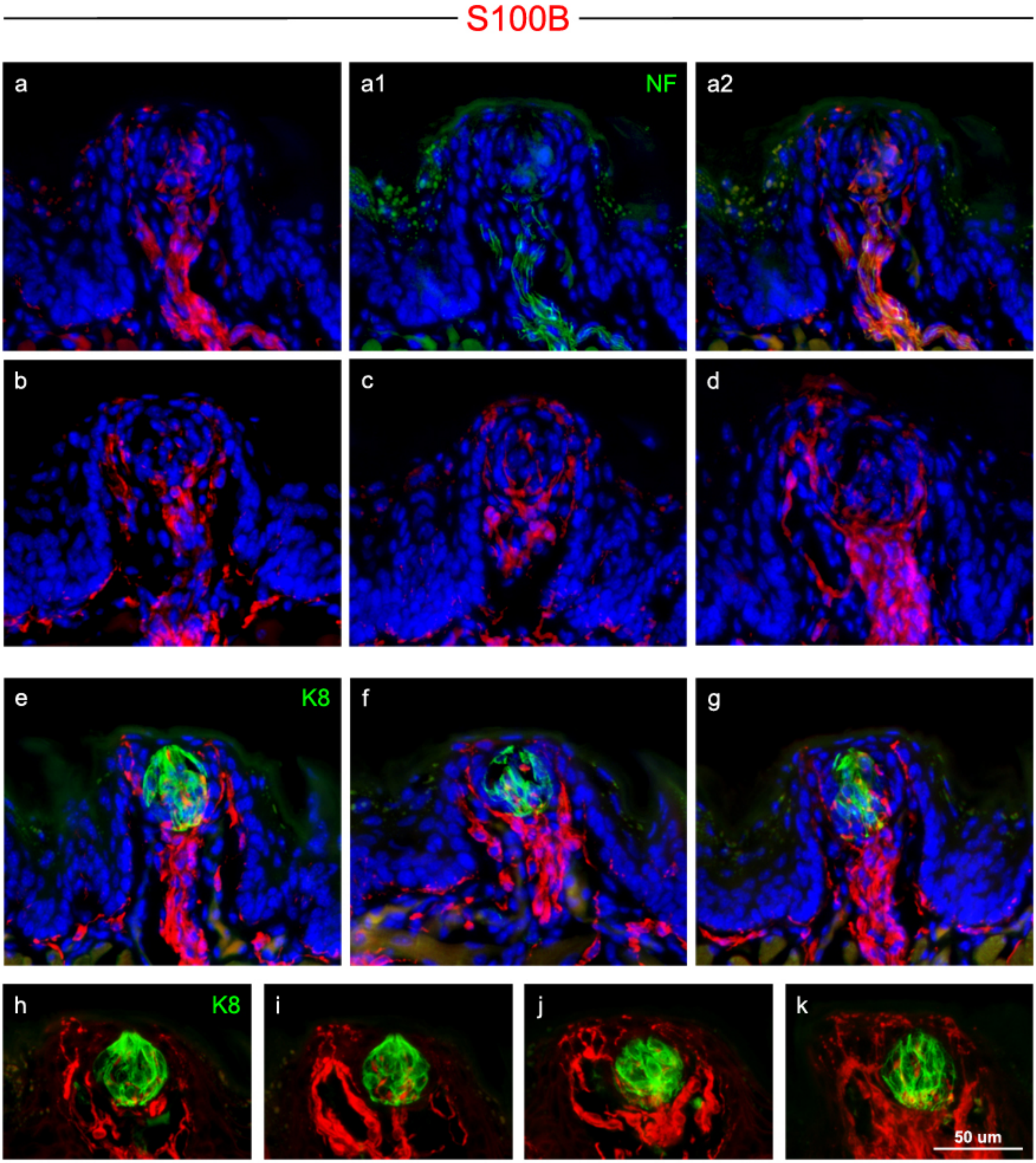

Figure S3

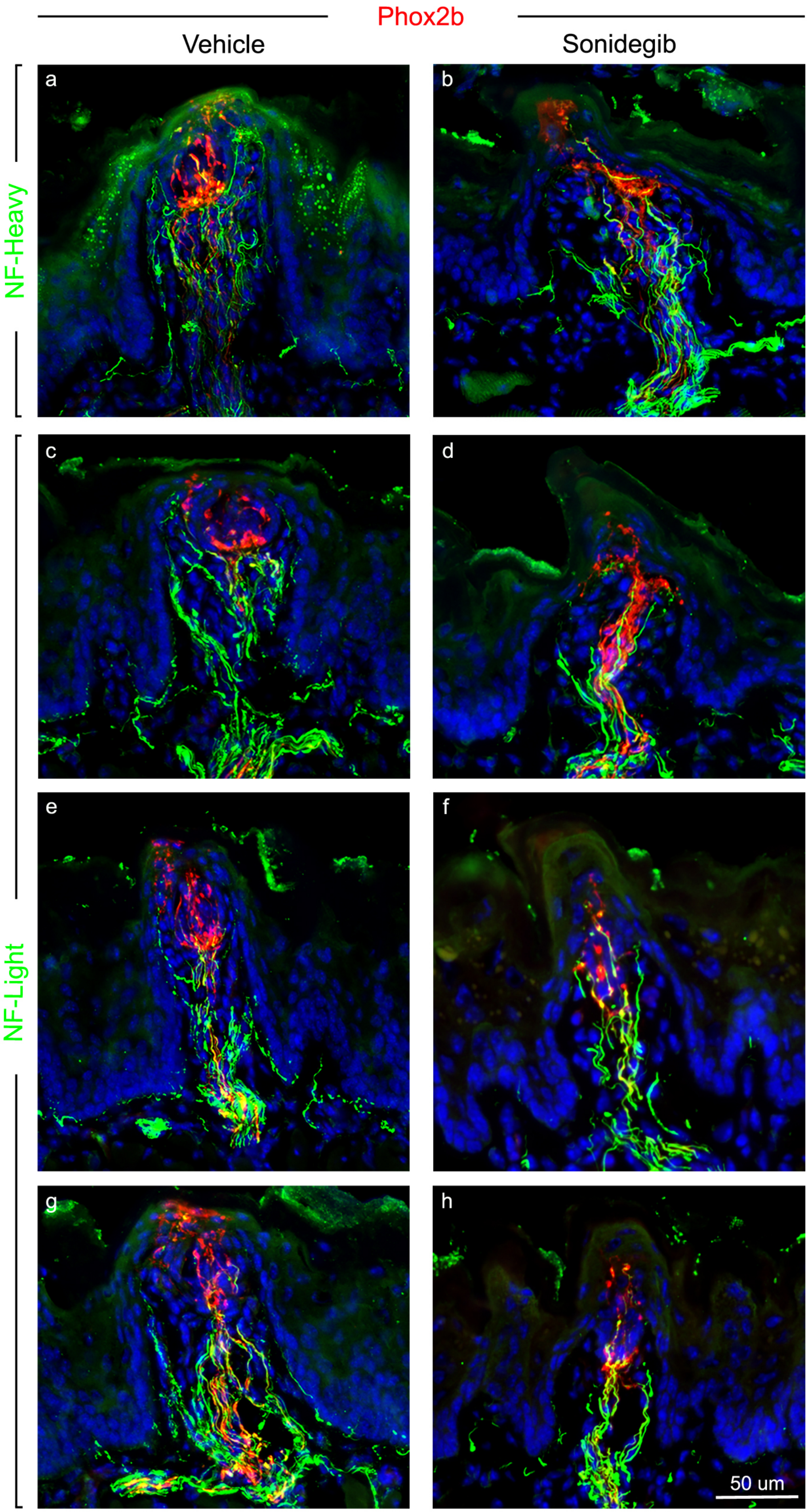

Figure S4

Homeostasis

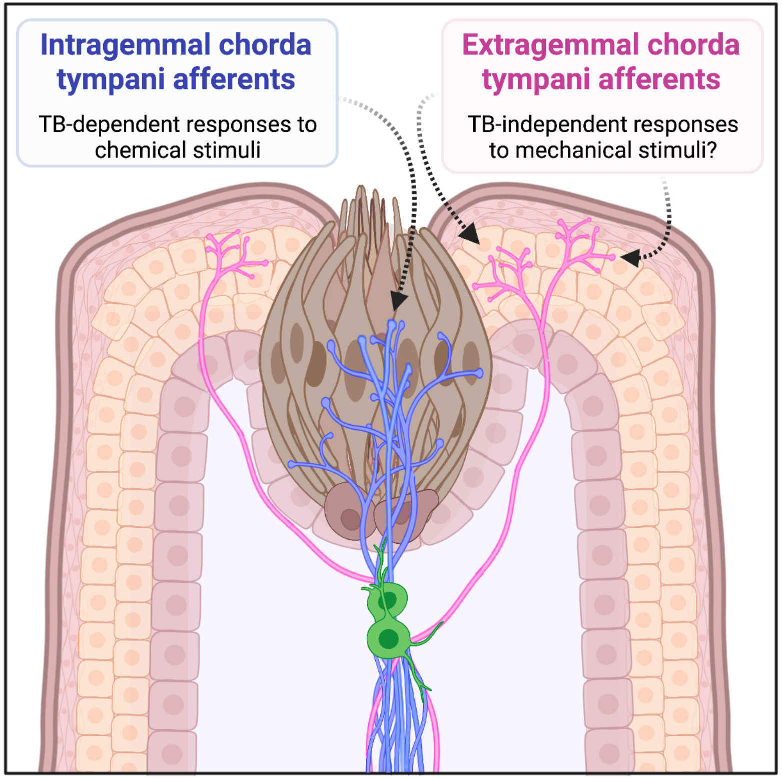

Chorda tympani-mediated responses to chemical, thermal, and mechanical stimuli

Hedgehog Pathway Inhibition

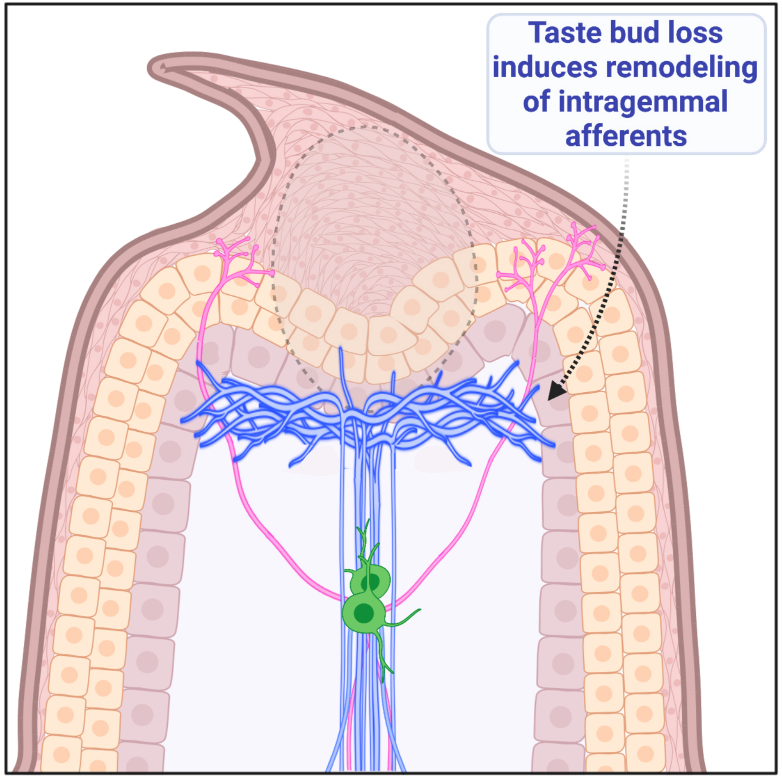

Chorda tympani-mediated responses to chemical stimuli lost; responses to mechanical stimuli maintained

Figure S5
